# Current gaps in management and timely referral of cardiorenal complications among people with type 2 diabetes mellitus in the Middle East and African countries: Expert recommendations

**DOI:** 10.1111/1753-0407.13266

**Published:** 2022-04-17

**Authors:** Alper Sonmez, Hani Sabbour, Akram Echtay, Abbas Mahdi Rahmah, Amani Matook Alhozali, Fahad Sulman al Sabaan, Fares H. Haddad, Hinde Iraqi, Ibrahim Elebrashy, Samir N. Assaad, Zaheer Bayat, Zeynep Osar Siva, Mohamed Hassanein

**Affiliations:** ^1^ Department of Endocrinology and Metabolism Gulhane School of Medicine, University of Health Sciences Ankara Turkey; ^2^ Heart & Vascular Institute Cleveland Clinic Abu Dhabi UAE; ^3^ Brown University Warren Alpert School of Medicine Providence Rhode Island USA; ^4^ School of Medicine Lebanese University Hadath Lebanon; ^5^ National Centre for Diabetes College of Medicine, Al‐Mustansriya University Baghdad Iraq; ^6^ Department of Medicine King Abdulaziz University Hospital Jeddah Saudi Arabia; ^7^ Security Forces Hospital Riyadh Saudi Arabia; ^8^ Endocrine & Diabetes, Abdali Hospital/Endocrine & Diabetes Clinic Amman Jordan; ^9^ Faculty of Medicine and Pharmacy Mohammed V University Rabat Morocco; ^10^ Faculty of Medicine Cairo University Cairo Egypt; ^11^ University of Alexandria Alexandria Egypt; ^12^ Division of Endocrinology and Metabolism, Department of Internal Medicine Helen Joseph Hospital Rossmore, Johannesburg South Africa; ^13^ Turkish Diabetes Association Turkey; ^14^ Dubai Hospital, DHA Dubai UAE; ^15^ Gulf Medical University Ajman UAE; ^16^ Cardiff University Cardiff UK

**Keywords:** cardiovascular risk management, diabetic cardiomyopathy, diabetic kidney disease, Middle East and North Africa and Africa, type 2 diabetes mellitus, 心血管风险管理, 糖尿病心肌病, 糖尿病肾病, 中东和非洲, 2型糖尿病

## Abstract

The upsurge of type 2 diabetes mellitus is a major public health concern in the Middle East and North Africa (MENA) and Africa (AFR) region, with cardiorenal complications (CRCs) being the predominant cause of premature morbidity and mortality. High prevalence of cardiometabolic risk factors, lack of awareness among patients and physicians, deficient infrastructure, and economic constraints lead to a cascade of CRCs at a significantly earlier age in MENA and AFR. In this review, we present consensus recommendations by experts in MENA and AFR, highlighting region‐specific challenges and potential solutions for management of CRCs. Health professionals who understand sociocultural barriers can significantly increase patient awareness and encourage health‐seeking behavior through simple educational tools. Increasing physician knowledge on early identification of CRCs and personalized treatment based on risk stratification, alongside optimum glycemic control, can mitigate therapeutic inertia. Early diagnosis of high‐risk people with regular and systematic monitoring of cardiorenal parameters, development of region‐specific care pathways for timely referral to specialists, followed by guideline‐recommended care with novel antidiabetics are imperative. Adherence to guideline‐recommended care can catalyze utilization of sodium glucose cotransporter 2 inhibitors and glucagon‐like peptide 1 receptor agonists with demonstrated cardiorenal benefits—thus paving the way for overcoming care gaps in a cost‐effective manner. Leveraging digital technology like electronic medical records can help generate real‐world data and provide insights on voids in adoption of newer antidiabetic medications. A patient‐centric approach, collaborative care among physicians from different specialties, alongside involvement of policy makers are key for improving patient outcomes and quality of care in MENA and AFR.

## Graphical abstract

**Highlights**

- This consensus document identifies challenging areas and strategic recommendations for the effective management of cardiorenal complications in type 2 diabetes mellitus in the Middle East and North Africa and Africa.
- There is a need for optimum glycemic control, early identification of cardiorenal complications, along with risk management through optimal low‐density lipoprotein cholesterol and blood pressure control, smoking cessation, and lifestyle management.
- Suboptimal use of international guidelines and treatment inertia delay initiation of adequate therapy.
- Early diagnosis of high‐risk people with regular and systematic monitoring of cardiorenal parameters, development of region‐specific care pathways for timely referral to specialists, followed by guideline‐recommended care with novel antidiabetics are imperative for optimal management.

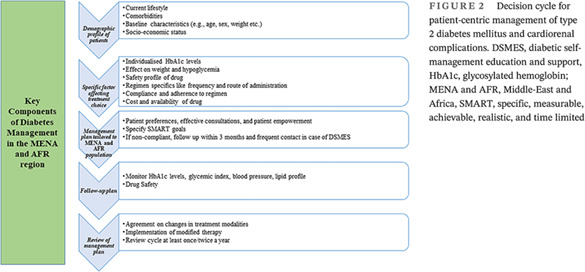

## 摘要

2型糖尿病是中东及北非(MENA)和非洲(AFR)地区的一大公共卫生问题，心肾并发症(CRC)是过早发病和死亡的主要原因。心脏代谢危险因素的高发、患者和医生缺乏认识、基础设施不足以及经济限制导致MENA和AFR患者的CRC年龄显著提前。在这篇综述中，我们提出了MENA和AFR地区专家的共识和建议，强调了区域特有的挑战和潜在的区域控制中心管理解决方案。了解社会文化障碍的卫生专业人员可以通过简单的教育工具显著提高患者的意识并鼓励寻求健康的行为。增加医生在早期识别CRC和基于风险分层的个性化治疗方面的知识，同时更好地控制血糖，从而减轻治疗的惰性。必须对高危人群进行早期诊断，对心肾功能进行定期和系统的监测，制定特定区域的护理路径，及时转诊至专家，然后根据指南建议的治疗，使用新型抗糖尿病药物。遵循指南推荐的治疗可以使用钠葡萄糖共转运体2抑制剂和胰高血糖素样肽1受体激动剂，它们已被证明对心肾有好处，从而以经济高效的方式为治疗技术的鸿沟铺平道路。利用数字技术，如电子病历，可以辅助生成真实世界的数据，以填补对较新的抗糖尿病药物效果观察的空白。以患者为中心的方法、不同专科医生之间的协作护理以及政策制定者的参与是改善MENA和AFR地区患者结局和治疗质量的关键。

## 1 INTRODUCTION

Type 2 diabetes mellitus (T2DM), affecting approximately 463 million people in 2019, is a global public health concern—particularly in low‐income and middle‐income countries (LMICs).^1^, ^2^ A conglomeration of modifiable risk factors including physical inactivity, unhealthy dietary patterns, increased blood pressure, dyslipidemia, and obesity, alongside nonmodifiable risk factors such as an aging population, family history of diabetes, and ethnicity are driving the current rise of T2DM and its complications in the Middle East and North Africa (MENA) and Africa (AFR) region.^3^ Of the top 20 countries with the highest prevalence of diabetes, 7 are from the MENA region.^4^

The ripple effect of persistent hyperglycemia and metabolic deviations in T2DM results in organ damage with different microvascular and macrovascular complications. Microvascular complications, comprising nephropathy, neuropathy, and retinopathy, along with macrovascular complications, including coronary artery disease, peripheral artery disease, and cerebrovascular disease, are the major cause of mortality and morbidity in patients with T2DM.^5^, ^6^ Indeed, cardiovascular (CV) complications such as myocardial infarction (MI), atrial fibrillation, and chronic heart failure (CHF) occur a decade earlier in the MENA and AFR region than in other populations.^7^, ^8^, ^9^ To prevent CV‐related mortality in patients with T2DM, optimum management of cardiorenal risk factors and complications is crucial, along with glycemic control. Screening programs for early detection of T2DM, compounded by timely utilization of safe and effective therapies, may help prevent complications, thus reducing morbidity and mortality.^6^, ^10^

The prevalence of cardiorenal complications in patients with T2DM is rising in the MENA and AFR regions. The cross‐sectional Turkish Diabetes Epidemiology (TURDEP)‐II survey involving 26 499 adults (≥20 years) showed an alarming rise in the prevalence of T2DM and associated cardiometabolic risk factors, highlighting an urgent need to address this major public health burden.^11^ Similarly the Dyslipidemia International Study (DYSIS)‐Middle East registry showed that 61.8% of very high‐risk patients (T2DM with atherosclerotic cardiovascular disease [ASCVD]) failed to achieve control of cardiometabolic risk factors.^12^ Moreover, the DYSIS‐II showed a progressive increase in the incidence of T2DM and simultaneously challenges in glycemic and cardiometabolic risk factor control in this growing population.^13^ To identify the current challenges for the management of cardiorenal complications in T2DM in MENA and AFR, we convened a panel of experts to provide insights on the regional gaps with strategic recommendations for treatment. In addition, we aimed to foster the development of real‐world evidence that can support patients with T2DM, encourage patient‐centric projects for cardiorenal complications, and improve patient outcomes.

## 2 CONSENSUS METHODOLOGY

A multidisciplinary panel of 13 members representing the specialties of endocrinology, cardiology, and internal medicine across the MENA and AFR region (Saudi Arabia = 2, United Arab Emirates [UAE] = 2, Egypt = 2, South Africa = 1, Lebanon = 1, Jordan = 1, Iraq = 1, Morocco = 1, and Turkey = 2) provided insights on practical challenges and recommendations to overcome the gaps in prevention and care. This manuscript is an outcome of literature review, expert group discussion, and consensus recommendations for the management of cardiorenal complications in patients with T2DM in MENA and AFR.

## 3 BURDEN AND ASSOCIATED RISK FACTORS FOR CARDIORENAL COMPLICATIONS IN MENA AND AFR

**FIGURE 1:**
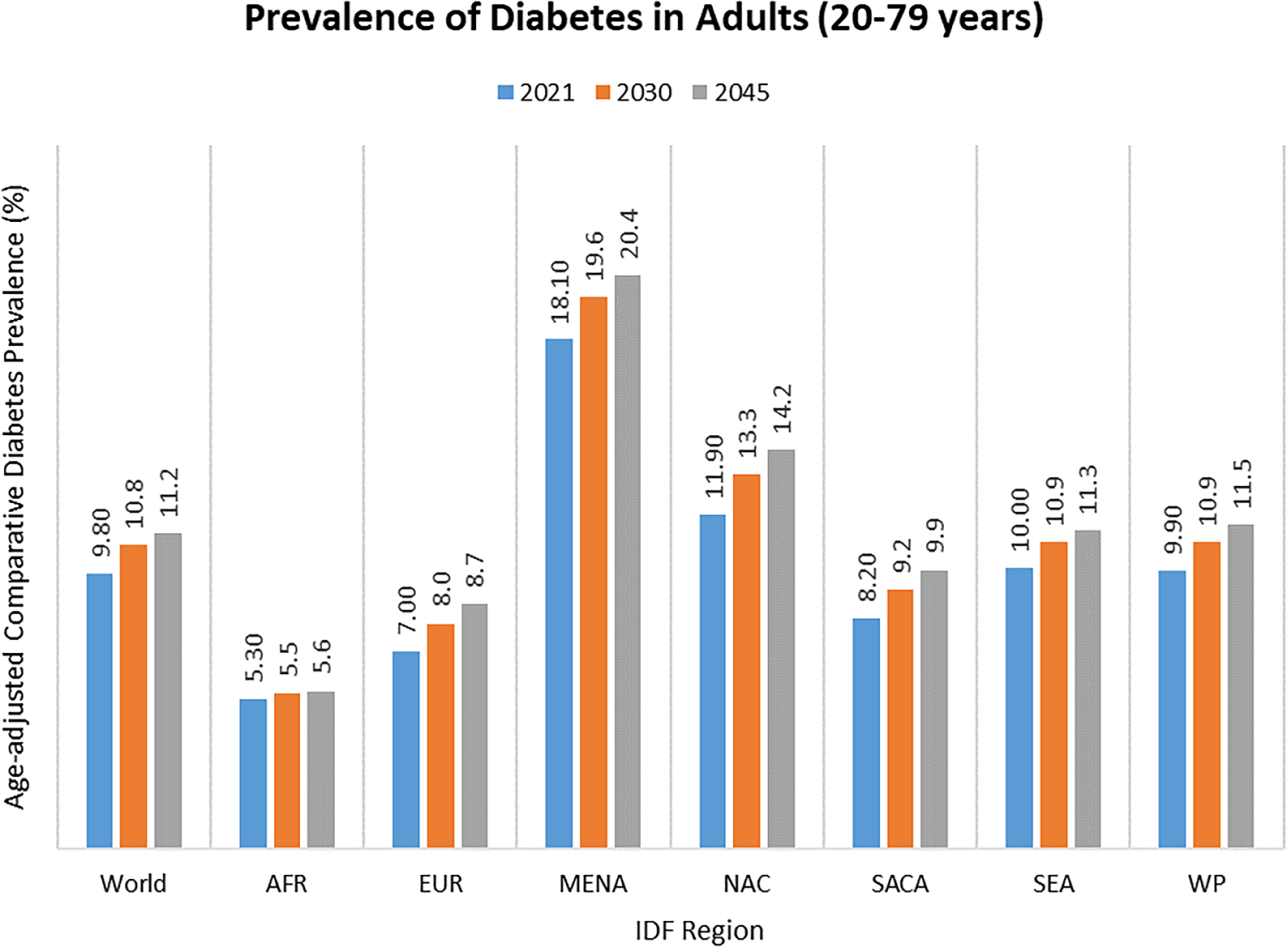
Prevalence of type 2 diabetes mellitus in the Middle East and Africa Region compared to the global scenario. AFR, Africa; EUR, Europe; IDF, International Diabetes Federation; MENA, Middle East and North Africa; NAC, North America and Caribbean; SACA, South and Central America; SEA, South‐East Asia; WP, Western Pacific^14^

Globally, the MENA and AFR region had the highest (18.1%) prevalence of T2DM in 2021 (Figure 1).^14^ The majority (90%) of people with T2DM in MENA and AFR are overweight or obese, with poor control of low‐density lipoprotein cholesterol levels, resulting in increased CV risk.^15^, ^16^ T2DM is a major risk factor for the development of cardiorenal complications—58% of individuals with T2DM develop chronic kidney disease (CKD), which concurrently increases the risk of heart failure (HF) and MI.^17^ The multicountry DISCOVER study among patients initiating a second‐line glucose‐lowering therapy reported that only 1 in 10 individuals in MENA and AFR had glycosylated hemoglobin (HbA1c) < 7.0%, alongside a high burden of cardiometabolic risk factors including hypertension and dyslipidemia.^18^ A similar trend in high cardiometabolic risk factors was reflected by the DISCOVER Global Registry.^19^ Cardiovascular registries from MENA and AFR, including but not limited to Gulf RACE (acute coronary syndrome [ACS]), Gulf Coast (ACS), and Gulf CARE (CHF), have consistently demonstrated the large burden of T2DM, obesity, hypertension, and dyslipidemia leading to premature cardiovascular disease (CVD) in spite of the young age of patients enrolled.^20^, ^21^, ^22^, ^23^ The DISCOVER program illustrated a substantial burden of microvascular complications (including CKD and albuminuria) and macrovascular complications (primarily coronary artery disease, HF, and stroke).^24^ Another multinational study (CAPTURE) showed that people with T2DM and established CVD (33%) have a higher burden of renal dysfunction (microalbuminuria [31.8% vs 20.6%], macroalbuminuria [10.8% vs 6.8%], and estimated glomerular filtration rate [eGFR] of ≤ 59 mL/min/1.73 m^2^ [30.7% vs 15.4%]) compared to those without CVD.^25^ CKD (44.3%) and CVD (17.3%) are the most prevalent comorbidities in T2DM.^26^ People with T2DM have almost quadruple the risk (hazard ratio [HR] 3.77, *P* = .001), while those with coronary heart disease are 2.47 (*P* = .004) times more likely to develop CKD stages 3‐5.^27^ A multinational registry of T2DM reported that cardiorenal disease was consistently the most frequent first manifestation (60%), stemming from 24% HF and 36% CKD, with HF being associated with a 2‐fold risk of CKD and vice versa.^28^ The combination of HF and CKD is associated with the highest CV risk (HR 3.91; 95% CI, 3.02‐5.07) and all‐cause mortality risk (HR 3.14; 95% CI, 2.90‐3.40).^28^ Table S1 provides an overview of the cardiorenal burden and associated risk factors in MENA and AFR.^9^, ^15^, ^16^, ^20^, ^21^, ^22^, ^23^, ^26^, ^27^, ^29^, ^30^, ^31^, ^32^, ^33^, ^34^, ^35^, ^36^ Reliable and consistent approaches are needed to measure routine cardiorenal parameters to guide clinical decision‐making in T2DM. Alongside the substantial burden, cardiorenal diseases incur the highest short‐ and long‐term health care costs in T2DM and are important parameters associated with increased health care resource utilization.^37^, ^38^ The Take CaRe of Me program has been initiated to create an end‐to‐end management model focusing on early prevention of cardiorenal complications of T2DM in the primary care setting.^39^ With the increasing prevalence of cardiorenal comorbidities in the MENA and AFR region, it is critical to identify the gaps and solutions for optimal management. Herein, we present the real‐world challenges and recommendations suggested by the experts for the management of cardiorenal complications in T2DM in the MENA and AFR region.

## 4 EXPERT DISCUSSION: CHALLENGES AND RECOMMENDATIONS IN MANAGEMENT OF CARDIORENAL COMPLICATIONS OF T2DM IN MENA AND AFR

### 4.1 Patient level

#### 4.1.1 Challenges

Lower education level of patients is an independent determinant of poor glycemic control in T2DM.^40^ There are voids in awareness pertaining to glycemic control, implications of suboptimal glycemic control, and subsequent cardiorenal complications of T2DM in most patients from MENA and AFR.^41^, ^42^, ^43^, ^44^ Poor access to diabetic educators or certified nurses, deficient infrastructure for patient education, and lack of educational tools are barriers for optimal care of T2DM in MENA and AFR.^45^, ^46^, ^47^ Sociocultural barriers such as preference for female educators by women, stigma related to the use of glucometers in public, and noncompliance with diet (likely during fasting in Ramadan) lead to challenges for appropriate management.

#### 4.1.2 Recommendations

Enhanced patient awareness of risk factors and early identification of diabetic complications are fundamental to improve quality of life. To this end, the development of simple and structured learning tools with education campaigns for physicians and patients can be crucial.^48^, ^49^ Due to variability in traditional sociocultural factors throughout the MENA and AFR region, risk assessment in primary care is warranted. Enhanced involvement of diabetic educators and nurses who understand the local sociocultural barriers can potentially influence patient engagement—thus encouraging frequent visits to educators for better care.^50^, ^51^

### 4.2 Physician level

#### 4.2.1 Challenges

Lack of awareness among primary care physicians (PCPs) and family doctors regarding the identification of risk factors for diabetic complications, screening asymptomatic cardiac and vascular disease, systematic screening for renal disease, and microalbuminuria in a standardized fashion remains a crucial challenge in MENA and AFR.^52^ Moreover, the use of antidiabetic medications for T2DM in the MENA and AFR region is driven by glycemic control, rather than cardiorenal risk reduction. As PCPs are the first‐contact care providers, awareness and knowledge of risk factors regarding cardiorenal complications in T2DM among PCPs are crucial. The majority of patients with T2DM are treated by generalists lacking expertise in treating such patients, leading to gaps in patient selection for the right antidiabetic medications.^47^ In addition, lack of systematic communication between different specialists, endocrinologists, cardiologists, or nephrologists, leading to a delay or nonreferral of patients to the specialists by PCPs further hinders T2DM management.^53^ Failure to reduce complexity and intensity of glycemic control in the older and frail population is another significant concern reported in 1 in 10 older adults with diabetes.^54^

- *Therapeutic inertia*: Barriers to appropriate care including therapeutic inertia appears to be an important contributor to poor glycemic control in MENA and AFR, with prescribers often willing to tolerate extended periods of “mild” hyperglycemia leading to delays in initiating or intensifying glucose‐lowering therapy when needed.^55^, ^56^, ^57^ Treatment inertia exposes patients with T2DM to long periods of raised blood glucose, predisposing them to complications and a reduced quality of life. Despite suboptimal metabolic control, almost half of the patients with T2DM were using injectable regimens in Turkey,^40^ with metformin, secretagogues, and dipeptidyl peptidase IV inhibitor (DPP‐IVi) being the most common oral antidiabetic drugs.^58^ The Saudi Arabia cohort of the DISCOVER study demonstrated that metformin (with or without sulfonylureas) was the most common first‐line treatment, while DPP‐IVis were the most common second‐line drugs for patients managed either in governmental institutions or in the private sector, irrespective of drug affordability. This underlines the presence of clinical inertia at the initiation of second‐line therapy, thus emphasizing a need for more aggressive risk factor screening with treatment intensification at early disease stages.^59^, ^60^, ^61^, ^62^, ^63^, ^64^ Although data on the novel antidiabetic drugs are still evolving, there is a gap between evidence‐based management suggested by international guidelines and real‐life practice in different regions.^65^, ^66^, ^67^, ^68^, ^69^, ^70^

#### 4.2.2 Recommendations

**FIGURE 2:**
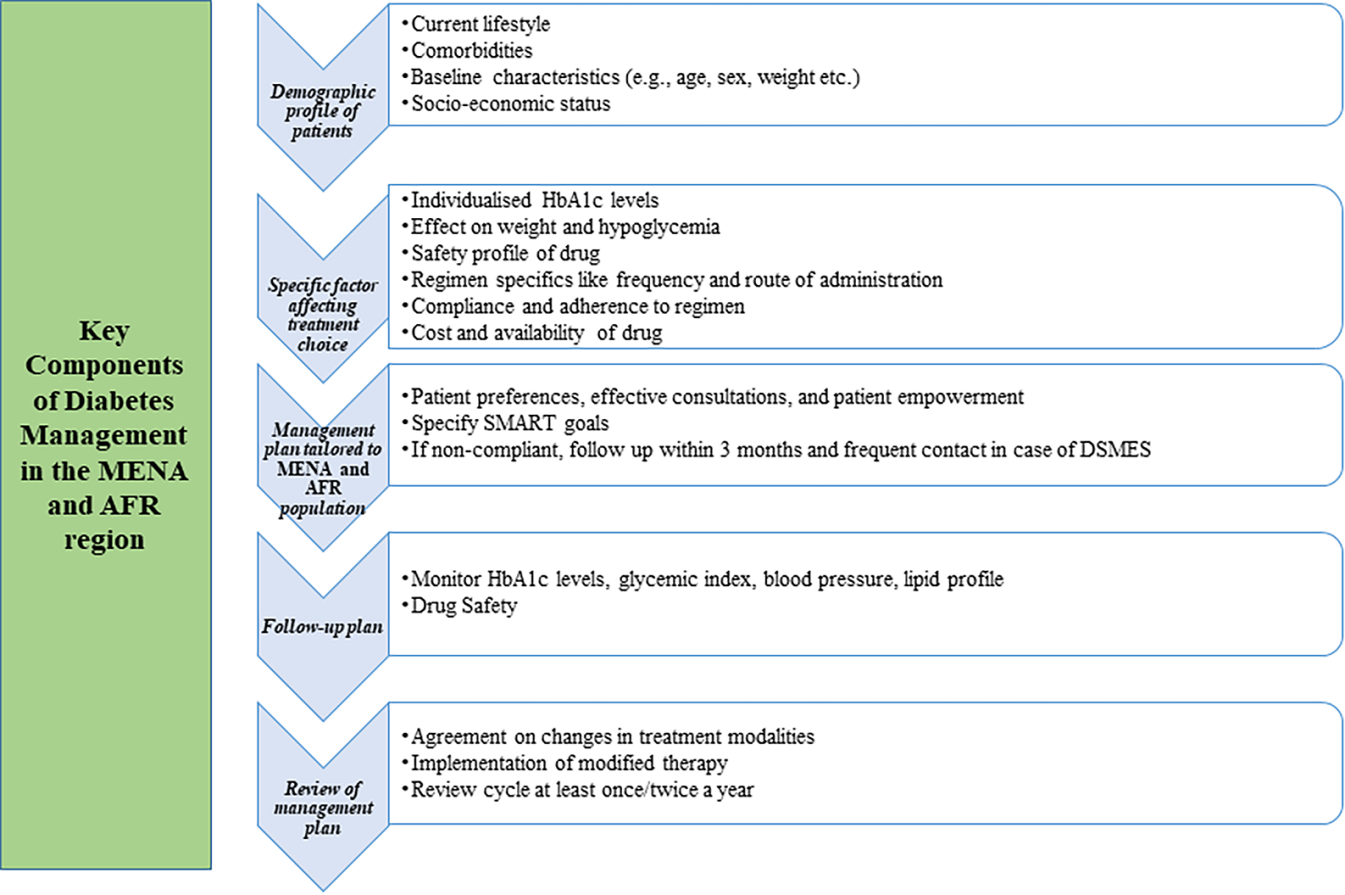
Decision cycle for patient‐centric management of type 2 diabetes mellitus and cardiorenal complications. DSMES, diabetic self‐management education and support, HbA1c, glycosylated hemoglobin; MENA and AFR, Middle‐East and Africa, SMART, specific, measurable, achievable, realistic, and time limited

It is imperative to shift the focus from a glycemic control‐centric approach to adopting a holistic risk‐based strategy, considering adverse effects and patient preferences. There is a critical need to address treatment inertia through early commencement of medications or escalation/de‐escalation, whenever required, to avert complications.^71^ Physicians should be well versed in the different types of pharmacotherapy for T2DM and select the accurate, effective, and safe drugs that patients can tolerate. Simplified treatment algorithms to recommend universal utilization of novel cardiorenal therapies in the earlier stage of diabetes is essential. Empowering nonphysician providers such as pharmacists, nurses, and diabetes educators to initiate and intensify treatment, supported by appropriate guidelines, might be an effective approach for mitigating therapeutic inertia.^72^ In Turkey, the *Consensus Statement of Endocrinology*, *Cardiology*, *and Nephrology (ENCARNE) Experts on Prevention*, *Diagnosis*, *and Management of Cardiovascular and Renal Complications of Diabetes* was formulated by collaborative body to provide a multidisciplinary platform for guiding PCPs through collective discussions on prevention, diagnosis, and management of cardiorenal complications, such as the need for referral to a specialist, frequency of visiting a specialist, and type and frequency of testing.^73^ Targeted screening using simple, sensitive, and cost‐effective tools can facilitate early identification and intervention of high‐risk populations, thus delaying the onset or progress of cardiorenal complications.^74^, ^75^ A patient‐centric approach for the management of T2DM and related complications is elucidated in Figure 2. Hence, continuous medical education of PCPs and family doctors on screening for T2DM, identifying complications, using guideline‐recommended antidiabetic medications, and addressing lifestyle and sociocultural issues is pivotal.^48^

- *Risk stratification and early diagnosis*: Risk stratification traditionally used for dyslipidemia management is now equally important for the selection of diabetic therapy in T2DM patients with three or more risk factors like obesity, smoking, older age, or hypertension and those with established target organ damage. Diagnosis of early complications of T2DM may require additional cardiac and renal investigations as well as assessment of target organ damage. ENCARNE illustrates diagnostic algorithms in T2D including assessment of serum creatinine, eGFR, urine albumin‐to‐creatinine ratio (UACR), and annual follow‐up for renal disease, alongside cardiac evaluation by electrocardiogram (ECG) every 1 or 2 years.^73^ Early diagnosis of left ventricular hypertrophy (LVH), nephropathy, retinopathy, and diabetic peripheral neuropathy is especially important in MENA and AFR. The earliest complications of T2DM including diastolic dysfunction, metabolic inflammatory cardiomyopathy, and HF with preserved ejection fraction can be effectively diagnosed with a combination of abnormal ECG and natriuretic peptide levels (B‐type natriuretic peptide [BNP] ≥35 pg/mL] or N‐terminal pro‐B‐type natriuretic peptide [NT‐proBNP] ≥125 pg/mL]).^76^ An echocardiogram should be requested in patients who present with new cardiac symptoms to assess silent cardiac abnormalities in the early stages. Assessment of UACR before progression to microalbuminuria and any abnormality in eGFR can predict early stage diabetic renal complications. Among T2DM patients with established complications, regular and systematic monitoring through ECG, UACR, eGFR, body mass index, fundus examination, and foot physical examination is imperative. Early diagnosis in an outpatient setting by PCPs can facilitate prompt addition of novel agents such as sodium glucose cotransporter 2 inhibitors (SGLT2is) and/or glucagon‐like peptide 1 receptor agonists (GLP‐1RAs) that can arrest the progression and symptomatology of the CHF phenotype and have an impact on normalization of eGFR with reduction of microalbuminuria at the earliest stages.
- *Novel cardiorenal therapies*: Novel antidiabetic agents like SGLT2is and GLP‐1RAs have been proven to have cardiorenal benefits in robust evidence‐based clinical trials, leading to their expansion to nondiabetic patients.^77^, ^78^, ^79^ A recent meta‐analysis showed that SGLT2is were associated with a reduced risk of major adverse cardiovascular events (MACE); the greatest benefit was a reduction in the risk of hospitalization for heart failure (HHF) and kidney outcomes.^80^ Similarly, GLP‐1RAs, have been reported to reduce the risk of individual MACE components, HHF, and worsening kidney function in T2DM.^81^, ^82^, ^83^ Dapagliflozin (compared with placebo) was associated with the greatest reduction in risk of developing composite kidney outcome, followed by empagliflozin, canagliflozin, semaglutide, and liraglutide.^84^ Tables S2‐S4 provide a broad overview of major randomized controlled trials, real‐world studies, and meta‐analyses for SGLT2is, GLP‐1RAs, and thiazolidinediones.^77^, ^79^, ^80^, ^82^, ^83^, ^85^, ^86^, ^87^, ^88^, ^89^, ^90^, ^91^, ^92^, ^93^, ^94^, ^95^, ^96^, ^97^, ^98^, ^99^, ^100^, ^101^, ^102^, ^103^, ^104^, ^105^, ^106^, ^107^, ^108^, ^109^, ^110^, ^111^, ^112^, ^113^, ^114^, ^115^, ^116^, ^117^, ^118^, ^119^, ^120^, ^121^, ^122^, ^123^, ^124^, ^125^, ^126^, ^127^, ^128^, ^129^, ^130^, ^131^, ^132^, ^133^, ^134^, ^135^, ^136^, ^137^, ^138^, ^139^, ^140^, ^141^, ^142^, ^143^, ^144^, ^145^ The benefits of dapagliflozin and empagliflozin that they lower the risk of CV events, HHF, and all‐cause mortality have been evidenced through real‐world studies in routine clinical care and nationwide registries.^111^, ^113^ Combination therapy is also a recommended clinical practice for improving glycemic control. The long‐term efficacy of insulin sensitizer thiazolidinedione (pioglitazone) plus a GLP‐1RA caused a greater decrease in HbA1c (−1.1%, *P* < .0001) and produced a greater improvement in insulin secretion with a lower risk of hypoglycemia in patients with poorly controlled T2DM.^106^ However, despite the CV benefits, pioglitazone should be used judiciously in clinical settings due to the definite increase in HF, bone fractures, weight gain, edema, and anemia.^145^
- *Utilization of guideline‐recommended therapy*: Based on cardiovascular outcome trials (CVOTs), recent guidelines strongly recommend the use of novel antidiabetic therapies for T2DM with high‐risk factors or established ASCVD, CKD, or HF. Traditionally, the American Diabetes Association (ADA) guidelines recommend metformin with lifestyle modifications as first‐line therapy.^65^ However, in patients with high‐risk factors or established cardiorenal disease, the ADA recommends the following strategies, independent of baseline HbA1c or individualized HbA1c target or metformin use: (a) ASCVD or indicators of high risk: either GLP‐1RA or SGLT2i with proven CVD benefit; (b) HF: SGLT2i with proven benefit (empagliflozin, canagliflozin, dapagliflozin); and (c) CKD: either GLP‐1RA or SGLT2i with proven benefits.^65^ The recent guidelines also recommend recognition of subclinical diabetic cardiomyopathy/CHF in people with early stage T2DM, CKD, or very high CV risk. A consistent approach was elaborated by the expert consensus recommendations in ENCARNE.^74^ The 2019 European Society of Cardiology (ESC) guidelines also recommend that patients with T2DM with either three or more major risk factors (T2DM, obesity, smoking, age, and hypertension) or established target organ damage (albuminuria, GFR, LVH, retinopathy, and neuropathy) may require SGLT2i or GLP‐1RAs, along with standard therapy.^66^ Adoption of early utilization of novel antidiabetic medication with metformin may be more practical in T2DM, particularly for addressing the management of glycemic control while simultaneously reducing cardiorenal and metabolic disorders. This approach may also overcome the problem of treatment inertia at the time of diagnosis, instead of progression to cardiorenal complications. Key recommendations from major guidelines for the management of T2DM and cardiorenal complications are illustrated in Table 1 and Table S2.^65^, ^66^, ^67^, ^68^, ^69^, ^82^, ^85^, ^86^, ^87^, ^88^, ^89^, ^90^, ^91^, ^92^, ^93^, ^94^, ^95^, ^96^, ^97^, ^98^, ^99^, ^100^, ^101^, ^102^, ^103^, ^104^, ^105^, ^106^, ^107^, ^108^, ^109^ The Dapagliflozin Effect on Cardiovascular Events‐Thrombolysis in Myocardial Infarction 58 (DECLARE‐TIMI 58) study has supported the expansion of SGLT2is for primary prevention of HF outcomes and reduction in atrial fibrillation.^87^ Evidence from the SGLT2is CVOTs lay the foundation for a change in focus from the traditional glycemic control to the reduction in cardiorenal complications in a cost‐effective manner.^146^

**TABLE 1:** Summarization of key recommendations from major guidelines for management of type 2 diabetes mellitus and cardiorenal complications

	*Screening*: Impaired glucose tolerance testing in asymptomatic individuals	*Glucose monitoring*: Self‐monitoring of blood glucose and/or continuous glucose monitoring	*Lifestyle*: Maintain body weight by adopting healthy diet and at least 150 min a week of moderate‐intensity physical activity	*Risk‐based therapy*: Personalized treatment based on hyperglycemia, diabetes duration, drug characteristics (efficacy, side effects, CV safety, contraindications, hypoglycemia risk, and cost), complications/comorbidities, life expectancy, patients' preferences	*Pathway of care*: Newly diagnosed T2DM: initial metformin with lifestyle modifications Established CV/CKD history: SGLT2i/GLP‐1RA^a^
International guidelines					
USA^65^	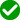 + Beginning at age 45 years; all overweight/obese adults with at least one risk factor at 3‐year interval; earlier and frequently with risk factors	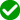	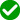 + Behavior change program to achieve/maintain 7% loss of initial body weight	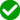	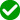 + Indicators of high‐risk/established ASCVD/CKD/HF: SGLT2i or GLP‐1RA, independent of baseline HbA1c/HbA1c target/metformin use Medication regimen and adherence should be reevaluated at regular intervals (3‐6 months) and adjusted as needed Treatment intensification based on 3‐6 monthly evaluation of drug regimen and adherence
Europe^66^	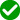 OGTT for diagnosing IGT Screening for T2DM in CVD patients T2DM patients to be screened annually for kidney disease	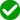	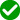	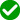	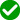 + Drug naïve: ASCVD, or high/very high CV risk (target organ damage or multiple risk factors): first‐line SGLT2i or GLP‐1RA monotherapy On metformin: ASCVD, or high/very high CV risk (target organ damage or multiple risk factors): add SGLT2i or GLP‐1RA
Regional guidelines					
Turkey^68^	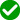 +Beginning at age 40 years, testing for all individuals with BMI ≥ 25 kg/m^2^ at 3‐year interval; earlier and frequently in presence of risk factors	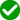	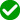	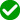	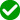
South Africa^69^	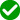 +Beginning at age 45 years, all overweight/obese adults with at least one risk factor; frequency depends on individual risk range (3 months to 3 years)	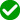	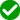	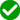	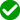 + GLP‐1RA/SGLT2i: Patients with established CV disease; to be managed at specialist care level Consider a SGLT2i as the third glucose‐lowering drug in those not achieving/maintaining glycemic targets on an oral two‐drug regimen GLP‐1RA injectable as the third drug (triple therapy) in overweight and obese patients when glycemic targets are not achieved or maintained
Emirates^67^	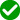 + Beginning at age 45 years, all adults with BMI ≥ 25 and ≥1 risk factor at least once every 3 years, or 6‐monthly if prediabetic	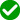	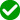	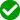	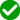 + Very high‐risk T2DM with CVD or target organ damage: GLP‐1RA or SGLT2i preferred as a second choice of treatment after metformin regardless of HbA1c level High‐risk T2DM without CVD or target organ damage: GLP‐1RA or SGLT2i preferred as second choice after metformin if HbA1c above target and if resources permit

Abbreviations: ASCVD, atherosclerotic cardiovascular disease; BMI, body mass index; CKD, chronic kidney disease; CV, cardiovascular; CVD, cardiovascular disease; GLP‐1RA, glucagon‐like peptide 1 receptor agonist; HbA1c, glycosylated hemoglobin; HF, heart failure; IGT, impaired glucose tolerance; OGTT, oral glucose tolerance test; SGLT2i, sodium glucose cotransporter 2 inhibitor; T2DM, type 2 diabetes mellitus.

a SGLT2is (empagliflozin, canagliflozin) or GLP‐1RAs (liraglutide, semaglutide).

## 5 HEALTH SYSTEM LEVEL

### 5.1 Challenges

The mean health care expenditure per person with diabetes complications per year in the UAE is more than US$5000.^4^ However the cost of medications is a small component of this. High cost and poor drug accessibility are among the many factors hindering health care. In MENA and AFR, insufficient access to novel medications in the public health sectors and the high cost of antidiabetic medications are the primary contributing reasons, together with country‐wise inequalities in health care financing, restricting the selection of a certain class of medications.^48^, ^147^, ^148^ The use of SGLT2is has been studied to be highly cost‐effective in preventing the cost of CV complications in LMICs.^149^, ^150^, ^151^

Shortage of regional data, poor adherence to international or regional guidelines, and inequalities in health care between private and public sectors are other challenges in the management of cardiorenal complications in MENA and AFR. Unavailability of medications in the public sector, insufficient care, and disparities of services contribute to patient flow to the private sector.^47^ Nearly 60% of patients with T2DM are not assessed by a specialist in MENA and AFR, with PCPs being the main first‐line treating physician.^152^, ^153^ Absence of electronic medical records (EMRs) may hinder sharing of patients' status and current therapies, leading to duplication of T2DM and other concomitant therapies. User resistance, lack of awareness, and gaps in strategic implementation are the common barriers for adopting EMRs in developing countries.^154^, ^155^ A survey‐based study of medical documentation in LMICs showed that the majority of the participating institutions used paper charting (64.2%) followed by institutional electronic health care record software (25.9%) for data entry during a patient visit.^156^ Capturing reliable patient data is challenging in most of the developing countries, including Iraq. The data collected during a routine patient visit may not be stable due to lack of attention to documentation and may hamper the physicians' ability to provide useful diagnosis to patients.^154^ Hence, it is imperative to emphasize the robust collection of physical patients records for diagnosis and prescribed drugs, followed by adequate maintenance at the physician and patient level in LMICs with gaps in systematic EMRs. As the world is transitioning to adopt the EMR system, with ongoing efforts toward making EMRs the preferred way of data collection, robust physical patient records can be the foundation stone for providing appropriate patient care in LMICs.

#### 5.1.1 Recommendations

Robust systems of EMRs along with the utilization of other novel digital technologies are needed to improve monitoring of glycemic control, the therapeutic landscape, and early detection of cardiorenal complications.^157^, ^158^ The panel shed light on the importance of adhering to international and regional management guidelines and suggested regulators to encourage the use of guidelines by general practitioners. Raising awareness of policy makers for enhanced utilization of novel antidiabetic drugs for preventing cardiorenal complications is central to T2DM management. Timely identification and resolution of barriers for utilization of guideline‐recommended therapies in clinical practice should be emphasized. In addition, the formulation of risk stratification tools tailored to different regions of MENA and AFR can be crucial to better inform treatment and policy‐level decisions. Patient support programs, advocacy groups, national diabetes registries, and national awareness campaigns are cornerstones for improving patient outcomes. Mandatory systemic audits, application of key performance indicators, and public health models to streamline the management process are recommended. Collaboration between different specialists, the establishment of cardiometabolic clinics to identify the differences in practice, and creating a simple questionnaire to assess the clinical need and track risk patterns among different specialists were considered essential to deliver the appropriate care and improve quality of life in T2DM. Additionally, assessment surveys at all levels of care with a subsequent formulation of regional cardiorenal guidelines should be endorsed by regulators and payers.

#### 5.1.2 Economic perspective

From a global public health perspective, implementation of the World Health Organization (WHO) Best Buys refers to cardiometabolic risk reduction in terms of treatment of diabetes, dyslipidemia, and hypertension.^159^ Most of the MENA region has been classified as a very high‐risk region based on the ESC risk stratification.^160^ The targeted use of CV and renal risk‐reducing SGLT2is and GLP‐1RAs has a clear public health implication, especially in the populations at higher CV risk.^161^ In addition, the involvement of national regulatory health authorities for the wider utilization of these novel antidiabetic medications has significant implications in the reduction of cardiorenal events in broad segments of the population. It also has significant health economic benefits as demonstrated in high‐income countries through reduction of downstream costs, such as hospitalization due to MI, HF, and renal replacement therapy. The cost‐effectiveness of the novel antidiabetic therapies, particularly SGLT2is, has been widely reported in the literature.^162^, ^163^, ^164^, ^165^, ^166^ The cost‐effective benefit is likely to have an even greater significance in resource‐limited countries, as utilization of complication‐preventing therapies would have an impact on population productivity and overall health care costs. Recent data including nationally representative surveys from 67 LMICs reported that using novel antidiabetic agents in a glycemia‐agnostic pathway produced a 92% reduction (SGLT2is) and 72% reduction (GLP‐1RA) in incremental cost‐effectiveness ratios in T2DM.^149^ The study elaborated that, consistent with the choice to include SGLT2is in the WHO Essential Medicines List, SGLT2is hold particular promise for reducing T2DM complications and meeting common price targets, particularly when used among people with established CV or kidney disease.

Challenges and key recommendations suggested at all levels—patient, physician, and health system—are tabulated in Table 2. Recognizing this urgent need for the creation of integrated cardiometabolic clinics in the UAE was highlighted in the long‐term multidisciplinary project UNITE inaugurated by the Emirates Diabetes Society and Emirates Cardiac Society (with collaboration from the American College of Cardiology [ACC] and ADA). This has resulted in the first cardiometabolic clinic in the region, which began operations in June 2021, and an official implementation pilot to the health care regulatory authorities for a population health initiative using this model of care. The growing prevalence of T2DM complications and the subsequent economic burden in the MENA region call for an urgent need and strategic response at all spheres of health care, including targeted interventions at the policy level.^167^, ^168^

**TABLE 2:** Challenges and key recommendations for management of type 2 diabetes mellitus and cardiorenal complications in the Middle East and Africa

	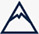 Challenges	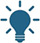 Recommendations
Patient level	Unfamiliarity with the asymptomatic stage of cardiovascular and renal disease Lack of access to educators and certified nurses Poor awareness about diabetes as a cause for cardiac and/or renal complications	Educators with knowledge of sociocultural barriers can utilize simple tools for patients' education and encourage health‐seeking behavior Enhance patient awareness on complications and impact on mortality and morbidity
Physician level	Unfamiliarity with systematic screening and identification of cardiorenal complications Treatment inertia Use of antidiabetic medications driven by glycemic control, rather than cardiorenal risk reduction Deferral in diagnosis and prevention of cardiorenal complications Difference in treatment patterns in the public and private sectors Dearth of communication between different specialists: endocrinologists, cardiologists, nephrologists Nonreferral of patients to the specialists by primary care physicians Suboptimal use of guideline‐recommended drugs by primary care physicians in clinical practice	Continuous medical education of primary care physicians and family doctors on novel antidiabetic medication use Education on screening and early identification of cardiorenal parameters Imperative to shift focus from a glycemic control‐centric approach to adopt a holistic risk‐based strategy, considering adverse effects and patient preferences Among T2DM patients with established complications, regular and systematic monitoring through ECG, UACR, eGFR, body mass index, fundus exam, foot physical exam is imperative Critical need to address treatment inertia through early commencement of medications or the escalation/de‐escalation, whenever required, to avert complications Regulators to encourage the use of guidelines by the general practitioners
Policy level	Unavailability of regional data Deficient infrastructure for patient education and deficient patient education tools Insufficiency of new class of medications in public health sector Unequal distribution of care between countries and intra‐country Shortfall of involvement of diabetic educators and nurses in care of patients with T2DM Country‐wise discrepancies in cost of medicines Unawareness of diabetes management guidelines Varied application of international guidelines	Develop simple, specific, sensitive, reproducible, and cost‐effective screening tools Establish population health models, diabetes national registries, cardiometabolic clinics, and awareness campaigns Convince policy makers for adopting new cardiorenal‐protective antidiabetic drugs Formulation of region‐specific guidelines for risk stratification, and appropriate care pathways with novel cost‐effective therapies Generation of real‐world data to identify voids in adoption of novel antidiabetic drugs

Abbreviations: ECG, electrocardiogram; eGFR, estimated glomerular filtration rate; T2DM, type 2 diabetes mellitus, UACR, urine albumin‐to‐creatinine ratio.

### 5.2 Policy shaping for effective care of T2DM and cardiorenal complications in MENA and AFR

With approximately one out of every seven people in MENA and AFR countries suffering from T2DM, coherent region‐wide approaches are warranted, alongside the currently existing short‐scale responses. Many countries such as UAE, Turkey, Oman, and South Africa have developed region‐specific guidelines for T2DM tailored to the needs of their population.^67^, ^68^, ^69^, ^70^ Predominantly targeting obesity, many countries have formulated programs such as reducing salt intake in food, raising taxes on tobacco and alcohol, or promoting public awareness for diet and physical activity.^169^ By 2019, 10 of the region's countries had policies relating to trans‐fatty acids, 13 countries had fully or partially implemented national salt reduction policies, and 8 countries had introduced taxes on carbonated or sugar‐sweetened beverages.^169^ With respect to diabetes care, Turkey has made steady progress undertaking initiatives such as the Diabetes Program of Turkey 2015‐2020 and the Multisectoral Action Plan of Turkey for Noncommunicable Diseases 2017‐2025, listing out priority approaches for strategic implementation.^170^, ^171^, ^172^ In the UAE, the Early Action in Diabetes policy, the “drive‐in” awareness‐raising initiative for improving quality of life of patients with T2DM, along with the Diabetes Prevention Program 2020 are noteworthy.^173^, ^174^ In Saudi Arabia, the Ministry of Health established specialized centers for the prevention, treatment, and rehabilitation of patients with T2DM through a network of integrated facilities. Other initiatives like the National Awareness Program for Diabetes 2013, the Antidiabetes Education National Program, and the National Executive Plan of Diabetes Control (2010‐2020) are crucial for primary and secondary prevention.^175^, ^176^ Countries like Oman and the UAE have initiated screening programs that can be instrumental for the early detection of noncommunicable diseases (NCDs).^177^ On the same lines, the Egypt National Multisectoral Action Plan for Noncommunicable Diseases 2017‐2021 was formed with emphasis on the following strategic areas: governance, risk reduction, health promotion, early detection and management, surveillance, monitoring and evaluation, and research.^178^ Similar initiatives for T2DM are ongoing in AFR with the aim to improve detection rates, address issues of lifestyle changes, and improve care monitoring and adherence to prescribed medicines.^42^

Notwithstanding the steady progress in policy shaping, only eight countries (Afghanistan, Islamic Republic of Iran, Kuwait, Saudi Arabia, Bahrain, Iraq, UAE, and Qatar) have an operational national strategy/action plan that integrates the major NCDs and their shared risk factors as of 2018.^179^ Only nine countries (Bahrain, Iran, Jordan, Kuwait, Lebanon, Oman, Saudi Arabia, UAE, and Palestine) have a provision of drug therapy and counseling to prevent CVD.^179^ Health expenditure on diabetes is still less in MENA and AFR countries, with a disparate standard of care across different regions.^180^ A recent pooled analysis from 55 LMICs, including MENA and AFR, reported that fewer than 1 in 10 people with T2DM receive coverage of guideline‐based comprehensive treatment.^181^ The study highlighted lower coverage for glucose‐lowering medication (50.5%), antihypertensive medication (41.3%), cholesterol‐lowering medication (6.3%), diet counseling (32.2%), exercise counseling (28.2%), and weight‐loss counseling (31.5%). Similarly, in AFR universal strengthening of the health system for better access to medicines for the management of cardiorenal complications, focusing on expanding the health technology assessment capabilities to assist with rational choices considering the pressure on resources, is warranted. Despite the formulation of several policies for T2DM in MENA and AFR, many are still in nascent stages of implementation in the real world, with gaps in the evaluation of key indicators for understanding their impact on the control of cardiorenal complications.^180^

### 5.3 Roadmap for driving patient‐centric diabetes care in MENA and AFR

Concerted efforts at a wider scale with better screening initiatives are required for effective T2DM care and prevention of cardiorenal complications in MENA and AFR. Higher investments to raise health care capabilities, particularly in the underserved primary care, can be pivotal to channel better diabetes management.^180^ Cross‐governmental approaches including different ministries, alongside engagement of the wider community can raise awareness and help navigate better patient‐centric care across the trajectory of T2DM. Implementation of chronic care models for T2DM and task‐sharing interventions with nonphysician health care workers can be instrumental in improving diabetes‐related outcomes.^182^, ^183^ Wider use of innovative digital technology, such as mobile health through telemedicine, may facilitate comprehensive treatment at a lower cost.^157^, ^184^ As chronic complications pose the biggest challenge, enhancing the capacity of health systems to deliver glucose‐lowering treatment and alongside addressing cardiorenal factors through timely and judicious use of novel antidiabetic agents with pleiotropic effects, are urgent priorities for diabetes care in the region.^181^

## 6 CONCLUSION

This consensus document identified challenging areas and outlined strategic recommendations for the effective management of cardiorenal complications in MENA and AFR. The use of antidiabetic medications for T2DM is driven by glycemic control rather than cardiorenal risk reduction. As PCPs are the first‐contact care providers, enhancing their knowledge on risk factors for cardiorenal complications and identification of high‐risk patients is crucial. Early diagnosis with regular and systematic monitoring of cardiorenal parameters, development of region‐specific care pathways for timely referral to a cardiologist or nephrologist, followed by guideline‐recommended care with novel antidiabetic agents are imperative. There is a critical need to address treatment inertia through early commencement of cardiorenal medications or the escalation/de‐escalation, whenever required, to avert complications. Early utilization of novel antidiabetic medication with metformin may be a pragmatic dual‐pronged approach for glycemic control while simultaneously reducing cardiorenal disorders in a cost‐effective manner, especially in resource‐limited countries. Despite the guideline recommendations, deficiencies in real‐world adoption of cardiorenal‐protective therapies should be identified and addressed for optimum management of cardiorenal complications in MENA and AFR.

## CONFLICT OF INTEREST

M.H., A.E., A.M.R., A.M.A., F.S.S., F.H.H., H.I., S.A., Z.B., and Z.O.S. declare no competing interests. A.S. reports participation in advisory boards of AstraZeneca, Novo Nordisk, Novartis, Eli Lilly, and Sanofi; meeting and travel support from AstraZeneca, Novo Nordisk, Amgen, and Sanofi; honoraria for educational lectures from Novo Nordisk, Boehringer Ingelheim, and Sanofi, and being the principal investigator in studies by Novo Nordisk and Novartis. I.E. reports participation in advisory boards of Abbott, AstraZeneca, Boehringer Ingelheim, Merck Sharp & Dohme, Novartis, Eli Lilly, Servier, Janssen, Novo Nordisk, Sanofi, EVA Pharma, Apex, and Amgen; participation as a speaker at AstraZeneca, Boehringer Ingelheim, Merck Sharp & Dohme, Novartis, Eli Lilly, Servier, Janssen, Merck Serono, Novo Nordisk, Sanofi, Amgen, EVA Pharma, Apex, hikma, Marcyrl, and Abbott; and participation in clinical trial research at Merck Sharp & Dohme, Novartis, Servier, and Novo Nordisk. H.S. reports honoraria for educational lectures and licenses from Novartis, AstraZeneca, Boehringer Ingelheim, Novo Nordisk, MAD, and Merck Sharp and Dohme. S.A. reports speaker honoraria from AstraZeneca, Boehringer Ingelheim, Merck Sharp and Dohme, Merk Serono, Novo Nordisk, Novartis, and Sanofi Aventis. The authors have no other relevant affiliations or financial involvement with any organization or entity with a financial interest in or financial conflict with the subject matter or editorial, research, and writing support for the development of the recommendations for gaps in the management of T2DM in the MENA and AFR region apart from those disclosed.

## Supporting information


**Table S1** Real‐world studies describing the burden and risk factors for cardiorenal complications in the Middle East and Africa region.
**Table S2**. Clinical trials describing cardioprotective and nephroprotective properties of major antidiabetic therapies.
**Table S3**. Real‐world studies describing cardioprotective and nephroprotective properties of major antidiabetic therapies.
**Table S4**. Meta‐analyses describing cardioprotective and nephroprotective properties of major antidiabetic therapies.
