# Supplementary material for: Current gaps in management and timely referral of cardiorenal complications among people with type 2 diabetes mellitus in the Middle East and African countries: Expert recommendations
Source: J Diabetes. 2022 Apr 17;14(5):315–33. doi: 10.1111/1753-0407.13266 (PMC9366572; doi:10.1111/1753-0407.13266)
Supplement: Supplementary file 1 — Table S1 Real‐world studies describing the burden and risk factors for cardiorenal complications in the Middle East and Africa region. Table S2. Clinical trials describing cardioprotective and nephroprotective properties of major antidiabetic therapies. Table S3. Real‐world studies describing cardioprotective and nephroprotective properties of major antidiabetic therapies. Table S4. Meta‐analyses describing cardioprotective and nephroprotective properties of major antidiabetic therapies. [file JDB-14-315-s001.docx]

**Supplementary Material**

**Supplementary Table 1: Real-world studies describing the burden and risk factors for cardiorenal complications in the Middle East and Africa region**

| Name of Study/Author | Design | Objective | Population Characteristics | Number of Patients | Treatment Modality | Outcome |
| --- | --- | --- | --- | --- | --- | --- |
| Gulf RACE^20^ | Prospective, observational study | Characteristics, management and hospital outcomes of ACS patients in the Gulf region of the Middle East. | Patients with the final diagnosis of ACS | 8176 | NA | - At presentation,   - 40% of patients had diabetes,   - 49% had hypertension,   - 32% had hyperlipidaemia,   - 38% were current smokers,   - 24% had a history of prior MI   - 41% had a history of prior angina. - Patients with STEMI [vs NSTEACS] were   - younger (54.3 ± 12.2 vs 57.23 ± 12.4 years; p<0.001)   - have higher rate of cigarette smoking (50% vs 28%; p<0.001). |
| Gulf RACE 2^21^ | Prospective, multicentre study | Clinical features, management,  in-hospital, and long-term outcomes in Arabian Gulf countries. | ACS patients with STEMI and NSTEACS, including non-STEMI and unstable angina | 7930 | NA | - The patients had history of   - CAD (57.3%)   - diabetes mellitus (39.5%)   - hypertension (47.2%)   - hyperlipidemia (32.7%). - Majority of patients were either current or ex-smokers (52.9%) and 18.4% were khat chewers. |
| Gulf Coast^22^ | Prospective, multinational longitudinal, observational, cohort-based registry | Risk profile, management and one-year outcomes of patients hospitalized with ACS in the Gulf region of the Middle East. | Patients   - - ≥18 years   - with ACS events | 3188 | NA | - The patients had previous history of   - hypertension (66%),   - dyslipidemia (55.8%),   - diabetes mellitus (53.4%) and   - current smoking (23.2%). |
| Gulf CARE^23^ | Prospective, multinational, multicentre registry | Clinical characteristics, management, and outcomes of acute HF patients from the Gulf acute heart failure registry (Gulf CARE) | Patients   - - above 18 year   - admitted to hospitals with the admission diagnosis of AHF | 5005 | NA | - The patients had previous history of   - hypertension (61%),   - diabetes mellitus (50%),   - CAD (47%),   - hyperlipidaemia (36%). - CAD was the most prevalent aetiology (53%) followed by idiopathic cardiomyopathy (18%), hypertensive heart disease (16%), and valvular heart disease (9%). |
| Al-Shami et al^29^ | Retrospective study | 9-year incidence rate of CVD and determine the risk factors associated with CVD among UAE nationals at high cardiovascular risk. | Patients   - - ≥18 year   - Without histories of CVD,   - who had ≥1 CVD risk factors | 977 | NA | - The patients had history of   - diabetes mellitus (42.8%),   - dyslipidemia (87.4%),   - hypertension (34.2%) and   - smoking (17.5%) and   - 47.3% had obesity. - Multivariable Cox analyses showed that   - total cholesterol-to- HDL-C ratio (HR, 1.44; 95% CI, 1.02 to 2.04) was an additional independent predictor of major CVD among women.   - age (HR, 1.50; 95% CI, 1.19 to 1.89) and a history of smoking (HR 1.80; 95% CI, 1.07 to 3.02) were significant risk factors associated with major CVD in men. |
| Kelishadi et al^30^ | Survey based study | Prevalence of overweight and obesity among Iranian adolescents and their relationship with modifiable environmental factors. | - Subjects, aged between 11 to 18 years | 2000 | NA | - A significant correlation was shown between BMI percentiles and serum triglyceride, HDL-C and systolic blood pressure (Pearson’s r = 0.38, –0.32 and 0.47, respectively). |
| PURE-Saudi^31^ | Prospective Urban Rural Epidemiology (PURE) study | Associated demographic, behavioral, and CVD risk factors as part of the PURE study. | Adults aged between 35 to 70 years | 2047 | NA | - Subjects had   - obesity (49.6%),   - dyslipidemia (32.1%),   - hypertension (30.3%),   - diabetes (25.1%) and   - 12.2% were current smokers. - Compared to participants in urban areas, a greater proportion of those living in rural areas had   - diabetes (31.1% rural vs 23.3% urban; p< 0.001),   - obesity (56.6% vs 47.3%; p< 0.001),   - hypertension (35.5% vs 28.6%; p = 0.004)   - low education level (46.4% vs 26.7%; p< 0.001)   - higher median BMI (30.8% vs 29.7%; p< 0.001). |
| CEPHEUS^15^ | Multicentre, non-interventional survey | Use of LLDs in patients with T2DM and co-existing dyslipidaemia. | Patients with T2DM taking LLD treatment for ≥3 months with no dose change for ≥6 weeks | 3338 | NA | - As per National Cholesterol Education Program Adult Treatment Panel III guidelines Out of 3338 patients,   - only 48% of the patients achieved their LDL-C goal   - 67.7% of the patients had a high CVD risk. - Of patients who achieved LDL-C goals (n=1589), nearly one-third were at very high CVD risk and the patients who had received statin monotherapy showed the highest proportion in LDL-C goal attainment, followed by those treated with fibrate monotherapy. - In a multivariate logistic regression model, taking drugs daily (OR: 1.64, 95% CI, 1.25 to 2.15) and older age (OR: 1.09; 95% CI, 1.01 to 1.18) were significantly associated with better odds of attaining LDL-C target. |
| Al-Jarallah et al^9^ | Prospective, multicenter, multinational registry | Mortality and morbidity in diabetic AHF patients stratified by left ventricular ejection fraction. | AHF patients had diabetes mellitus | 2258 | NA | - The patients had   - CAD (n=1658; 73.4%),   - hypertension (n=1843; 81.6%),   - dyslipidemia (n=1228; 54.4%),   - atrial fibrillation (n=274; 12.1%),   - chronic kidney disease (CKD; n= 525; 23.3%). - HFrEF (EF, <40%) was observed in 1268 patients (56.2%), 515 patients (22.8%) had HFmrEF (EF, 40–49%) and 475 patients (21.0%) HFpEF (EF, 50%). |
| Al-Ozairi et al^26^ | Cross-sectional multi-center observational study | Prevalence and co-prevalence of comorbidities, including CKD and CVD (CAD, cerebrovascular disease, PAD and CHF) among patients with T2DM. | Patients with T2DM | 300 | NA | - Most prevalent comorbidities were   - CVD (17.3%),   - CAD (15%),   - CKD (44.3%), mostly stages 2 and 3,   - CHF (0.7%),   - PAD (2.3%),   - cerebrovascular diseases (1.3%) - Half of the patients had T2DM only with no associated CVD or CKD (n=151), 38% had one comorbidity (n=113), 10% had two comorbidities (n=29), and 2% had three comorbidities (n=7) in addition to T2DM. - The number of comorbidities significantly increased with age (p < 0.001). |
| Afsharian et al^32^ | Population-based prospective study | Risk factors for CVD and mortality events in patients with T2DM and to calculate their population attributable fraction among a representative Iranian population. | Patients with T2DM | 1198 | NA | - Significant predictors for CVD events were   - FPG level of 7.22 to <10 mmol/L (HR, 1.46; 95% CI, 1.12 to 1.96; p = 0.011],   - FPG level ≥10 mmol/L (HR, 2.04; 95% CI, 1.53 to 2.72; p<0.0001),   - hypertension (HR, 1.65; 95% CI, 1.28 to 2.13; p<0.0001),   - hypercholesterolemia (HR, 1.96; 95% CI, 1.40 to 2.75; p<0.0001),   - high waist-to-hip ratio (HR, 1.30; 95% CI, 0.99 to 1.70; p = 0.051),   - age (HR, 1.04; 95% CI, 1.02 to 1.05; p<0.0001). - Significant predictors for all-cause mortality events were   - hypertension (HR, 1.70; 95% CI, 1.23 to 2.36; p = 0.001),   - FPG level ≥10 mmol/L (HR, 2.31; 95% CI, 1.55 to 3.20; p = 0.000),   - smoking (HR, 1.45; 95% CI, 1.03 to 2.04; p = 0.033) |
| Omar et al^33^ | Gulf DiabCare survey | Status of diabetes control and complications, and the quality of diabetes management in Saudi Arabia, Kuwait, and the UAE, and to obtain an insight into the relationship between these factors. | Patients with diabetes for >12 months | 1290 | NA | - Diabetes-related complications were   - neuropathy (34.9%),   - background retinopathy (29.9%),   - cataract (14.1%),   - cardiovascular complications (<10%)   - microalbuminuria (34.4%). |
| IDMPS study^34^ | Cross-sectional study | Management of care of patients in Africa with T2DM in current medical practice | Patients with T2DM | 3191 | NA | - Diabetes-related complications were present in 47.2% (n=1102)   - Majority of patients had microvascular complications (n=978; 41.9%),   - 267 patients (11.4%) had at least one documented macrovascular disease. |
| Jelinek et al^35^ | Prospective cross-sectional study | Clinical profiles of patients with T2DM in the UAE, including patterns, frequencies, and risk factors of microvascular and macrovascular complications. | Patients with T2DM | 490 | NA | - Most common T2DM comorbidities were   - hypertension (83.40%),   - obesity (90.49%),   - dyslipidemia (93.43%). - Additionally, 90.49% were either overweight or obese (mean BMI = 31.9 kg/m2, mean waist circumference >107 cm in men and >104 cm in women). - Significant predictor risk factors for the development of T2DM complications were   - duration of T2DM (OR, 1.66; 95% CI, 1.19 to 2.31; p = 0.003),   - levels of eGFR (OR, 0.97; 95% CI, 0.94 to 0.99; p = 0.010),   - total cholesterol (OR, 0.67; 95% CI, 0.48 to 0.94; p = 0.019). |
| Al-Shamsi, Regmi, and Govender^27^ | Retrospective study | Incidence and causes of CKD stages 3-5 in adult UAE nationals with or at high risk of CVD. | Patients with either CVD or a high CVD risk | 491 | NA | - Almost half of the patients had history of DM (215/491; 43.8%) and close to two-thirds had hypertension (335/491; 68.2%). - Male subjects (vs female) had higher prevalence of   - coronary heart disease (CHD; 13.2% vs 5.0%),   - vascular disease (8.8% vs 2.9%),   - smoking (29.2% vs 0.8%), - Female subjects (vs male) had higher prevalence of obesity (61.0% vs 40.4%). - Patients with a history of CHD were 2.47 (95% CI, 1.34 to 4.56; p = 0.004) times more likely to develop CKD stages 3–5 than those with no history of CHD. - Patients with diabetes had almost quadruple the risk (HR, 3.77; 95% CI, 1.79–7.96; p = 0.001) compared with those without diabetes. - History of smoking increased the risk of CKD stages 3–5 by 2.35 times compared with no history of smoking (95% CI, 1.27–4.34; p = 0.006). |
| TEMD OBESITY ^16^ | Cross-sectional, nationwide, multicenter survey | Prevalence of overweight and obesity among patients with T2DM and to search for the impact of obesity on the metabolic control of these patients |  | 4648 | NA | - Out of 4648, only 10.1% (n = 468) of the patients had a normal weight. - Both obesity (53.4 vs. 40.0%) and severe obesity (16.6 vs. 3.3%) are more prevalent in female patients with T2DM compared to male patients (p < 0.001) - Multivariate analysis hosed that the independent associates of obesity in patients with T2DM were being   - female (OR, 2.43; 95% CI, 2.12 to 2.78; p < 0.001),   - older age (OR 0.60; 95% CI, 0.42 to 0.86; p = 0.005 for ages 60–69 years; OR, 0.36; 95% CI, 0.25 to 0.53; p < 0.001 for age > 70 years),   - smoking (OR, 0.69; 95% CI, 0.57 to 0.84; p < 0.001),   - having microvascular complications (OR, 1.18; 95% CI, 1.03 to 1.35; p = 0.020),   - hypertension (OR, 1.74; 95% CI, 1.35 to 2.23; p<0.001),   - low HDL-C levels (OR, 1.31; 95% CI, 1.15 to 1.50; p<0.001) |
| Al-Rubeaan et al^36^ | Saudi National Diabetes Registry | Better understanding and accurate assessment of this chronic complication and its related risk factors | Patients with T2DM aged ≥25 years | 54670 | NA | - Overall prevalence of diabetic nephropathy was 10.8% (1.2% microalbuminuria, 8.1%macroalbuninuria and 1.5% end stage renal disease). - The risk factors for microalbuminuria were   - hyperlipidemia (relative risk [RR], 3.37; 95% CI, 2.74 to 4.15; p<0.0001),   - hypertension (RR, 2.4; 95% CI, 1.94 to 2.98; p<0.0001),   - retinopathy (RR, 1.66; 95% CI, 1.31 to 2.10; p<0.0001),   - neuropathy (RR, 1.62; 95% CI, 1.28 to 2.04; p<0.0001)   - obesity (RR, 1.43; 95% CI, 1.03 to 1.97; p = 0.003) |

ACS = acute coronary syndrome; AHF = acute heart failure; BMI = body mass index; CAD = coronary artery disease; CHD = coronary heart disease; CHF = congestive heart failure; CI = confidence interval; CKD = chronic kidney disease; CVD = cardiovascular disease; EF = ejection fraction; eGFR = estimated glomerular filtration rate; FPG = fasting plasma glucose; HDL-C = high-density lipoprotein-cholesterol; HF = heart failure; HFmEF = HF mid-range ejection fraction; HFpEF = heart failure with preserved ejection fraction; HFrEF = heart failure with reduced ejection fraction; HR = hazard ratio; KCCQ = Kansas City Cardiomyopathy Questionnaire score; LDL-C = low density lipoprotein cholesterol; LLD = lipid lowering drug; NA = not applicable; NSTEACS = non-ST-segment elevation acute coronary syndrome; OR = odds ratio; PAD = peripheral arterial disease; RR = relative risk; STEMI = ST-segment elevation myocardial infarction; T2DM = type 2 diabetes mellitus; UAE = United Arab Emirates

**Supplementary Table 2: Clinical trials describing cardioprotective and nephroprotective properties of major antidiabetic therapies**

| Drug Class | Name of Study/Author | Design | Objective | Population Characteristics | Number of Patients | Treatment Modality | Outcome |
| --- | --- | --- | --- | --- | --- | --- | --- |
| SGLT2i | EMPA‑REG OUTCOME^85^ | Randomized, double‑blind, placebo‑controlled study | Effect of once‑daily empagliflozin vs placebo on CV events | Patients with   - T2DM - established ASCVD‑99% | 7020 | Empagliflozin 10 mg vs Empagliflozin 25 mg vs placebo | - Empagliflozin group (vs placebo) had lower percentage of death from CV causes, nonfatal MI, or nonfatal stroke (10.5% vs 12.1%; HR; 0.86; 95% CI, 0.74 to 0.99; *p*<0.001 for noninferiority and *p*= 0.04 for superiority) . |
|  | CANVAS and CANVAS‑R^86^ | Randomized, single‑blind, placebo‑controlled study | Effect of canagliflozin on CV, renal, and safety outcomes | Patients with   - T2DM - established ASCVD‑65% | 10,142  (CANVAS:4330, CANVAS‑R:5812) | CANVAS (1:1:1) – canagliflozin: 300 mg vs  CANVAS (1:1:1) – canagliflozin: 100 mg vs matching placebo  CANVAS‑R (1:1) – canagliflozin: 100 mg with an optional increase to 300 mg vs matched placebo | - Canagliflozin group (vs placebo) had lower composite of death from CV causes, nonfatal MI, or nonfatal stroke (26.9 vs. 31.5 participants per 1000 patient-years; HR, 0.86; 95% CI, 0.75 to 0.97; *p*<0.001 for noninferiority; *p* = 0.02 for superiority). |
|  | DECLARE‑TIMI v58^87,88^ | Randomized, double‑blind, multinational, placebo‑controlled, Phase 3 study | Effect of dapagliflozin on CV outcomes | Patients with established ASCVD‑40% | 17,160 | Dapagliflozin 10 mg vs matched placebo (1:1) | - Dapagliflozin (vs placebo) resulted in a lower rate of cardiovascular death or HHF (4.9% vs. 5.8%; HR, 0.83; 95% CI, 0.73 to 0.95; p = 0.005) (DECLARE-TIMI-58, 2019). - Dapagliflozin (vs placebo) (DECLARE-TIMI-58, 2021)   - reduced risk of CV death or HHF (HR, 0.84; 95% CI, 0.67 to 1.04) and renal-specific outcome (HR, 0.51; 95% CI, 0.37 to 0.69).   - lowered HBA_1C_, eGFR, SBP and urinary albumin-to-creatinine ratio (p<0.001). |
|  | DAPA‑CKD^89^ | International, multicenter, randomized, double-blind, parallel group, placebo-controlled study | Effect of dapagliflozin on renal outcomes and CV mortality in patients with CKD | Patients with   - CKD - with or without T2DM | 4304 | Dapagliflozin 10 mg vs placebo | - Dapagliflozin group (vs placebo) had lower   - occurrence of composite of a sustained decline in the eGFR of at least 50%, ESRD), or death from renal or CV (9.2% vs 14.5%; HR, 0.61; 95% CI, 0.51 to 0.72; P<0.001; number needed to treat to prevent one primary outcome event, 19; 95% CI, 15 to 27),   - occurrence of death (4.7% vs 6.8%; HR, 0.69; 95% CI, 0.53 to 0.88; P=0.004).   - risk of composite of a sustained decline in the eGFR of at least 50%, end-stage kidney disease, or death from renal causes (HR, 0.56; 95% CI, 0.45 to 0.68, P<0.001) - risk for the composite of death from CV causes or HHF (HR, 0.71; 95% CI, 0.55 to 0.92; P = 0.009). |
|  | CREDENCE^90^ | Randomized, double-blind, event-driven, placebo-controlled, multicenter study | Effects of canagliflozin on renal and CV outcomes in participants with diabetic nephropathy | Patients with   - T2DM - diabetic nephropathy | 4401 | Canagliflozin 100 mg vs placebo | - Canagliflozin group (vs placebo) had 30% lower relative risk of composite of ESRD (dialysis, transplantation, or a sustained eGFR of <15 ml/minute/1.73 m2), a doubling of the serum creatinine level, or death from renal or CV causes with event rates of 43.2 and 61.2 per 1000 patient‑years, respectively (HR, 0.70; 95% CI, 0.59 to 0.82; P=0.0001). - Canagliflozin group (vs placebo) had lower   - relative risk of the renal-specific composite of ESRD, a doubling of the creatinine level, or death from renal causes by 34% (HR, 0.66; 95% CI, 0.53 to 0.81; P<0.001; P=0.002)   - relative risk of ESRD by 32% (HR, 0.68; 95% CI, 0.54 to 0.86; P=0.01),   - risk of CV death, MI, or stroke (HR, 0.80; 95% CI, 0.67 to 0.95) and HHF (HR, 0.61; 95% CI, 0.47 to 0.80; p<0.001). |
|  | DAPA‑HF^91^ | Randomized, placebo‑controlled, Phase 3 study | Effect of dapagliflozin on the incidence of worsening HF or CV death | Patients with   - established HF - HFrEF - with or without T2DM | 4744 | Dapagliflozin 10 mg vs placebo once daily | - Dapagliflozin group (vs placebo) had lower   - occurrence of composite of worsening HF or CV death (16.3% vs 21.2%; HR, 0.74; 95% CI, 0.65 to 0.85; p<0.001),   - first worsening HR event (10.0% vs 13.7%; HR, 0.70; 95% CI, 0.59 to 0.83),   - occurrence of death from CV (9.6% vs 11.5%; HR, 0.82; 95% CI, 0.69 to 0.98), - death from any cause (11.6% vs 13.9%; HR, 0.83; 95% CI, 0.71 to 0.97). |
|  | VERTIS^92^ | Randomized, double‑blind, placebo‑controlled study | CV outcomes with ertugliflozin in subjects with T2DM and established atherosclerotic cardiovascular disease | Patients with   - T2DM - established ASCVD | 8246 | Ertugliflozin 5 mg vs ertugliflozin 15 mg vs matched placebo once daily (1:1:1) | - Ertugliflozin group had MACE occurrence of 653 of 5493 patients (11.9%) compared to 327/2745 patients (11.9%) in the placebo group (HR, 0.97; 95.6% CI, 0.85 to 1.11, p<0.001). - Ertugliflozin group (vs placebo) had lower risk of   - death from CV causes was (HR, 0.92; 95.8% CI, 0.77 to 1.11),   - death from renal causes, renal replacement therapy, or doubling of the serum creatinine level was (HR, 0.81; 95.8% CI, 0.63 to 1.04). |
|  | EMPEROR‑Reduced^93^ | Randomized, event-driven double‑blind, parallel‑group, placebo‑controlled study | Efficacy and safety of once daily empagliflozin | Patients with chronic HFrEF | 3730 | Empagliflozin 10 mg vs placebo | - Empagliflozin group (vs placebo) had   - lower occurrence of composite of adjudicated CV death or HHF (19.4% vs 24.7%; HR,0.75; 95% CI, 0.65 to 0.86, p<0.001),   - lower total number of HHF (HF, 0.70; 95% CI, 0.58 to 0.85, p<0.001)   - slower annual rate of decline in the estimated glomerular filtration rate (-0.55 vs. -2.28 ml per minute per 1.73 m2 of body-surface area per year, P<0.001). |
|  | EMPERIAL^94^ | Randomized clinical trial | Effects of empagliflozin on exercise ability and patient-reported outcomes in HFrEF and HFpEF with and without T2DM, reporting, for the first time, the effects of SGLT-2i in HFpEF | Patients with   - HFrEF (≤40%, EMPERIA-Reduced) - HFpEF (>40%, EMPERIAL-Preserved) - with and without T2D | EMPERIAL-Reduced: 312; EMPERIAL-Preserved: 315 | Empagliflozin 10 mg vs placebo | - Empagliflozin group (vs placebo) had   - improvement in congestion score, and diuretic use and KCCQ-TSS responder rates,   - similar 6-minute walk test distance change to Week 12 (-4.0 m; (95% CI, -16.0 to 6.0; p = 0.42 vs. 4.0 m, 95% CI, -5.0 to 13.0; p = 0.37). |
|  | TQT study^95^ | Randomized, placebo-controlled, single-dose, double-blind, five-period crossover study | Assess potential effects of empagliflozin on ventricular repolarisation and other electrocardiogram (ECG) parameters. | Health volunteer of   - both sexes, - aged between ≥18 and ≤55 Y - BMI of ≥18.5 kg/m2 and ≤29.9 kg/m2 | 30 | Empagliflozin 25 mg vs empagliflozin 200 mg vs matched placebo vs moxifloxacin 400 mg (positive control, open-label) | - Empagliflozin 25 mg and 200 mg administration (vs placebo) did not induce clinically relevant prolongation of the mean QTcN interval after 1–4 hours administration (empagliflozin 25 mg: placebo-corrected MCfB, 0.6; 90% CI, -0.7 to 1.9 and empagliflozin 200 mg: placebo-corrected MCfB, -0.2; 90% CI, -1.4 to 0.9) ms). |
|  | DEFINE-HF Trial^96^ | Investigator-initiated, multi-center, randomized controlled trial | Test the hypothesis that treatment with the SGLT-2i dapagliflozin will improve natriuretic peptides and health status in well-phenotyped and optimally treated HFrEF patients, both with and without T2DM. | HF patients with   - LVEF ≤40% - NYHA class II-III - estimated glomerular filtration rate ≥30 mL/min/1.73m - elevated NT-proBNP with/ without T2DM | 263 | Dapagliflozin 10 mg vs matching placebo | - A greater proportion of patients treated with dapagliflozin (vs placebo) had a clinically meaningful improvement of ≥5 points in KCCQ-OS or at least a 20% reduction in NT-proBNP (61.5% vs 50.4%; adjusted OR, 1.8; 95% CI, 1.03 to 3.06, nominal p = 0.039). - In patients treated with dapagliflozin (vs placebo), at 12 weeks   - mean KCCQ-OS was 3.7 points higher (p = 0.037),   - mean KCCQ-CS was 4.6 points higher (p = 0.007). |
|  | EMPA-HEART^97^ | Double-blind, placebo-controlled, randomized investigator-initiated clinical trial | Determine if empagliflozin causes a decrease in LV mass in people with T2DM and coronary artery disease. | Patients   - aged ≥40 and ≤80 years - with glycated hemoglobin 6.5% to 10.0%, - with known coronary artery disease, - with eGFR of ≥60mL/min/1.73m‑ | 97 | Empagliflozin 10 mg/day vs placebo | - In empagliflozin group   - mean LV mass indexed to body surface area regression over 6 months was 2.6 g/m2 compared to 0.01 g/m2 in placebo group (adjusted difference −3.35 g/m2; 95% CI, −5.9 to −0.81g/m2, P=0.01),   - there was significant lowering of overall ambulatory systolic blood pressure (adjusted difference −6.8mmHg, 95% CI −11.2 to −2.3mmHg, P=0.003), diastolic blood pressure (adjusted difference −3.2mmHg; 95% CI, −5.8 to −0.6mmHg, P=0.02) and elevation of hematocrit (P=0.0003) compared to placebo. |
| GLP-1 RA | HARMONY^98^ | Double‑blind, randomized, placebo‑controlled study | Safety and efficacy of albiglutide in preventing CV death, MI, or stroke | Patients with   - T2DM - aged ≥40 Y   with CVD | 9463 | Albiglutide 30-50 mg vs matched placebo (1:1) | - Albiglutide group (vs placebo) had lower occurrence of   - first occurrence of death from CV causes, nonfatal MI, or nonfatal (7% vs 9%),   - incidence rate (4.6 events per 100 person‑years vs 5.9 events per 100 person‑years; HR, 0.78; 95% CI, 0.68 to 0.90; p<0.0001 for non-inferiority and p=0.0006 for superiority). - Albiglutide was superior to placebo (p<0.0001 for non-inferiority and p = 0.0006 for superiority). |
|  | EXSCEL^99^ | Randomized, double‑blind, placebo‑controlled study | CV outcomes after treatment with exenatide once weekly in patients with T2DM | Patients with established ASCVD‑73% | 14752 | Exenatide 2 mg vs matched placebo (1:1) | - Exenatide group (vs placebo) had lower occurrence of   - first occurrence of death from CV causes, nonfatal MI, or nonfatal (11.4% vs 12.2%),   - incidence rate (3.7 events per 100 person‑years vs 4.0 events per 100 person‑years; HR, 0.91; 95% CI, 0.83 to 1.00),   - similar efficacy (P=0.06). |
|  | LEADER^100^ | Randomized, double‑blind, placebo‑controlled study | Liraglutide effects on CV events | Patients with established ASCVD‑81% | 9340 | Liraglutide 1.8 mg vs placebo | - Liraglutide group (vs placebo) had lower   - first occurrence of death from CV causes, nonfatal MI, or nonfatal stroke (13.0% vs 14.9%; HR, 0.87; 95% CI, 0.78 to 0.97; p<0.001 for noninferiority; p = 0.01 for superiority),   - rate of death from any cause (8.2% vs 9.6%; HR, 0.85; 95% CI, 0.74 to 0.97; p = 0.02). |
|  | REWIND^82^ | Multicenter, randomized, double‑blind, placebo‑controlled study | Assess if dulaglutide can reduce major CV events and other serious outcomes in persons with T2DM, when added to their anti‑hyperglycemic regimen | Patients with established ASCVD‑31% | 9901 | Dulaglutide 1.5 mg vs placebo (1:1) | - Dulaglutide group (vs placebo) had lower   - primary composite outcome (12·0% vs 13·4%),   - incidence rate (2·4 vs 2·7 per 100 person-years; HR, 0·88; 95% CI 0·79 to 0·99; p = 0.026). |
|  | PIONEER 6^101^ | Randomized, event-driven double‑blind, placebo‑controlled study | CV safety of oral semaglutide in subjects with T2DM | Patients with T2DM at high CV risk   - age of ≥50 Y - with established CVD or CKD - age of ≥60 Y with CV risk factors only | 3183 | Oral semaglutide 14 mg daily once vs placebo | - Oral semaglutide group (vs placebo) had lower occurrence of MACE (3.8% vs. 4.8%; HR, 0.79; 95% CI, 0.57 to 1.11; p<0.001). |
|  | SUSTAIN‑6^102^ | Long‑term, randomized, double‑blind, placebo‑controlled, multinational, multi‑center study | CV and other long‑term outcomes with semaglutide in subjects with T2DM | Patients with established ASCVD‑83% | 3297 | Semaglutide 0.5 mg vs semaglutide 1.0 mg vs volume‑matched placebo (1:1:1:1) | - Semaglutide group (vs placebo) had lower first occurrence of CV death, nonfatal MI, or nonfatal stroke (6.6% vs 8.9%; HR, 0.74; 95% CI, 0.58 to 0.95; p<0.001). |
|  | ELIXA^103^ | Randomized, double‑blind, placebo‑controlled, parallel‑group, multicenter Study | CV outcomes during treatment with lixisenatide in patients with T2DM after an acute coronary syndrome | Patients with T2DM,   - had a MI   hospitalized for unstable angina within the previous 180 days | 6068 | Lixisenatide 10 µg per day to a maximum dose of 20 μg vs volume matched placebo | - Lixisenatide (vs placebo) addition to usual care did not alter the rate of MACE or other serious adverse events. |
|  | [Tonneijck](https://pubmed.ncbi.nlm.nih.gov/?size=20&term=Tonneijck+L&cauthor_id=29915021) et al^104^ | Randomized, open label, comparator-controlled, parallel-group, intervention trial | Compare the effect of prolonged lixisenatide treatment with insulin glulisine with respect to blood pressure, insulin concentrations, arterial stiffness and systemic vascular resistance. | Overweight insulin glargine-treated patients with T2DM | 34 | Lixisenatide 20 μg or titrated insulin glulisine once-daily | - Lixisenatide (vs insulin glulisine) increased   - systemic vascular resistance (P<0.001) and arterial stiffness (P=0.007),   - SBP by 5.2±2.9 mm Hg (maximal difference, +10.2±3.7 mm Hg; P=0.087),   - DBP by 5.4±1.4 mm Hg (maximal difference, +7.2±1.5 mm Hg; P<0.001). |
| Thiazolidinedione | Satirapoj et al^105^ | Randomized, open labeled, controlled study | Efficacy and side effects of low dose pioglitazone with standard dose pioglitazone among patients with T2DM and CKD. | Patients with   - T2DM - CKD | 75 | Pioglitazone 7.5 and 15 mg/day | - Pioglitazone 7.5 and 15 mg/day resulted in no serious AEs related or unrelated to pioglitazone including drug-induced hepatotoxicity, severe hypoglycemia and congestive HF. |
|  | Qatar Study^106,107^ | Open label, single-center, randomized control trial | Efficacy of combination therapy with exenatide plus pioglitazone vs basal/bolus insulin in patients with long-standing poorly controlled T2DM on metformin plus a sulfonylurea | Patients with   - T2DM - poorly controlled HbA1c >7.5%, 58 mmol/mol | 231 (2017)  331 (2020) | - Combination therapy, pioglitazone (15 mg/day for 2 week and increased to 30 mg/day at week 4) and exenatide (2 mg/week extended-release) vs basal (glargine)/bolus insulin therapy | - Combination therapy group (vs insulin therapy) [Qatar study, 2017]   - had decreased HbA1C (0.7%, P < 0.0001),   - more subjects achieved ADA treatment goal (HbA1c <7.0%; 83% vs. 53%, P = 0.003), - Insulin therapy group (vs combination therapy) experienced greater weight gain (2.1 ± 1.1 vs. 4.2 ± 1.0 kg, p < 0.0001) and a threefold higher rate of hypoglycemia (6.6 vs. 2.3 events per patient-year; p<0.0001) [Qatar study, 2017]. - Combination therapy group (vs insulin therapy) [Qatar study, 2020]   - caused grater decrement in HbA1C (-1.1%, P < 0.0001),   - more subjects achieved ADA treatment goal (HbA1c <7.0%; 86% vs. 44%, P < 0.001). - Insulin therapy group (vs combination therapy) experienced greater weight gain (9.6 ± 1.5 vs. 5.2 ± 1.3 kg; p= 0.0003) and 2.5 fold higher rate of hypoglycemia (0.28 vs 0.12 events per patient month; p<0.0001) [Qatar study, 2020]. |
|  | IRIS^108^ | Double-blind, placebo-controlled randomized clinical trial | Association between baseline characteristics, post-randomization events (including MI), study drug side effect reports, and dose adjustments, on the development of HF | Patients with   - IR - without T2DM | 3851 | Pioglitazone 15 mg daily and increased to 30 mg at 4 weeks and 45 mg at 8 weeks vs placebo | - Pioglitazone (vs placebo) reduced the composite outcome of stroke, MI or hospitalized HF (HR, 0.78; p=0.007). |
|  | TOSCA.IT^109^ | Multicentre, randomized, pragmatic clinical trial | Long-term effects of pioglitazone versus sulfonylureas, given in addition to metformin, on CV events in patients with T2DM | Patients with   - T2DM - aged 50-75 years - uncontrolled with metformin monotherapy | Pioglitazon: 1535; sulfonylureas: 1493 | Metformin monotherapy (2-3 g per day) and add-on pioglitazone, 15-45 mg vs sulfonylurea (5-15 mg glibenclamide/ 2-6 mg glimepiride/ 30-120 mg gliclazide) | - Pioglitazone (vs sulfonylurea)   - lower episodes of hypoglycemias (10% vs 34%, p<0.0001),   - had similar risk of composite of first occurrence of all-cause death, non-fatal MI, non-fatal stroke, or urgent coronary revascularisation (HR, 0·96; 95% CI 0·74-1·26; p = 0.79). |

ADA = American Diabetes Association; AEs = adverse events; ASCVD = atherosclerotic cardiovascular disease; BMI = body mass index; CI = confidence interval; CKD = chronic kidney disease; CV = cardiovascular; CVD = cardiovascular disease; DBP = diastolic blood pressure; ESRD = end‑stage renal disease; eGFR = estimated glomerular filtration rate; GLP-1 RA = glucagon-like peptide-1 receptor agonists; HbA_1C_ = glycated hemoglobin; HF = heart failure; HHF = hospitalization for heart failure; HFpEF = heart failure with preserved ejection fraction; HFrEF = heart failure with reduced ejection fraction; HR = hazard ratio; IQR = interquartile range; IR: insulin resistance; KCCQ-CS = Kansas City Cardiomyopathy Questionnaire clinical summary score; KCCQ-OS = Kansas City Cardiomyopathy Questionnaire overall summary score; KCCQ-TSS = Kansas City Cardiomyopathy Questionnaire total symptom score; LV = left ventricular; LVD = left ventricular dysfunction; LVEF = left ventricular ejection fraction MACE = major adverse cardiovascular events; MI = myocardial infraction; MCfB = mean change from baseline; NA = not applicable; NT-proBNP = N-terminal pro b-type natriuretic peptide; NYHA = New York Heart Association; OR = odds ratio; SGLT-2i = sodium-glucose co-transporter 2 inhibitor; SBP = systolic blood pressure; T2DM = type 2 diabetes mellitus

**Supplementary Table 3. Real-world studies describing cardioprotective and nephroprotective properties of major antidiabetic therapies**

| **Drug class** | **Name of study/Author** | **Design** | **Objective** | **Population Characteristics** | **Number of Patients** | **Description of treatment** | **Outcome** |
| --- | --- | --- | --- | --- | --- | --- | --- |
| **SGLT2i** | CVD-REAL Study^110^ | PS matched study | Compare HHF and death in patients newly initiated on any SGLT2i versus other GLD in 6 countries real-world setting | Patients with   - T2DM   Newly started on either SGLT-2i or other GLD | SGLT2i, 154 528; other GLD, 154 528 | NA | - Use of SGLT2i (vs other GLD) was associated with lower rates of   - HHF (hazard ratio, 0.61; 95% CI, 0.51–0.73; P<0.001),   - death (hazard ratio, 0.49; 95% CI, 0.41–0.57; P<0.001),   - HHF or death (hazard ratio, 0.54; 95% CI, 0.48–0.60; P<0.001). |
|  | CVD-REAL Nordic^111^ | 1:3 PS matched study | Investigate associations of hospitalization for kidney disease  (HKD), HFF and all-cause death between new users of either dapagliflozin or DPP-4i in T2D patients | Patients with T2DM | Dapagliflozin, 8582; DPP-4i, 25746 | NA | - Dapagliflozin (vs DPP-4i) use was associated with lower risk of   - HKD (HR, 0.38; 95% CI, 0.29 to 0.51; p <0.001),   - HHF (HR, 0.63; 95% CI, 0.50 to 0.81; p <0.001),   - all-cause death (HR, 0.73; 95% CI, 0.59 to 0.91; p = 0.004). |
|  | CVD-REAL 2 Study^112^ | PS matched study | Examine a broad range of CV outcomes in patients initiated on SGLT-2i versus other GLDs across 6 countries in the Asia Pacific, the Middle East, and North American regions | Patients with   - T2DM either initiated on SGLT-2i or other GLD | SGLT2i, 235,064; other GLD, 235,064 | NA | - Use of SGLT2i (vs other GLD) was associated with lower rates of   - HHF (HR, 0.64; 95% CI, 0.50 to 0.82; p = 0.001),   - death (hazard ratio, 0.51; 95% CI, 0.37 to 0.70; P<0.001),   - HHF or death (HR, 0.60; 95% CI, 0.47 to 0.76; p<0.001),   - MI (HR, 0.81; 95% CI, 0.74 to 0.88; p<0.001),   - stroke (HR: 0.68, 95% CI, 0.55 to 0.84; p<0.001). |
|  | EMPRISE^113^ | 1:1 PS matched cohort study | Assess risk of HHF among patients with T2DM initiating empagliflozin vs. sitagliptin. | Patients with   - T2DM, - aged ≥18 years - initiating empagliflozin or sitagliptin | 32886 | Empagliflozin 10 mg or 25 mg per day vs sitagliptin | - Empagliflozin initiation (vs. sitagliptin)   - decreased the risk of HHF-specific by 50% (HR=0.50; 95% CI, 0.28 to 0.91),   - decreased the risk of HHF-broad by 49% (HR=0.51; 95% CI, 0.39 to 0.68),   - lower incidence of HHF-specific (2.1 vs. 6.7) and HHF-broad (10.5 vs. 22.2) outcomes were observed in empagliflozin PS matched initiators. |
|  | Patorno et al^114^ | Population based retrospective cohort study | CV safety of canagliflozin, in direct comparisons with DPP-4i, GLP-1 RA, or sulfonylureas, as used in routine practice | Patients with T2DM | First cohort: canagliflozin, 21 431; DPP-4i, 77 463  Second Cohort: canagliflozin, 25 806; GLP-1RA, 32 676  Third cohort: canagliflozin, 18 924; sulfonylurea, 115 435  PS-matched cohorts: canagliflozin or DPP-4i, 17 667 pairs; canagliflozin or GLP-1RA, 20 539; canagliflozin or sulfonylurea, 17 354 | NA | - Canagliflozin was associated with lower risk of HF admission to hospital with   - HR, 0.70 (95% CI, 0.54 to 0.92) vs DPP-4i (n=17 667 pairs),   - HR, 0.61 (95% CI, 0.47 to 0.78) vs GLP-1 RA (n=20 539),   - HR, 0.51 (95% CI, 0.38 to 0.67) vs. sulfonylurea (n=17 354). - canagliflozin was associated with   - lower risk of composite CV endpoint (HR, 0.89, 95% CI, 0.68 to 1.17) vs DPP-4i   - lower risk of composite CV endpoint (HR, 0.86; 95% CI, 0.65 to 1.13) vs sulfonylurea   - higher risk of composite CV endpoint (HR, 1.03; 95% CI, 0.79 to 1.35) vs GLP-1 RA. |
|  | Birkeland et al^115^ | 1:1 PS matched study | Compare the new use of sodium-glucose SGLT2i versus DPP-4i and the risk of cardiorenal disease, HF or CKD in patients with T2DM without a history of prevalent cardiovascular and renal disease | Patients with   - T2DM   Without history of prevalent CV and renal disease | SGLT2i, 105130;  DPP-4i, 105130 | NA | - SGLT2i (vs DPP-4i) was associated with lower risk of   - cardiorenal disease (HR, 0.56; 95% CI, 0.42 to 0.74),   - HF (HR, 0.71; 95% CI, 0.59 to 0.86),   - CKD (HR, 0.44; 95% CI, 0.28 to 0.69),   - all-cause mortality disease (HR, 0.67; 95% CI, 0.59 to 0.77),   - cardiovascular mortality (HR, 0.61; 95% CI, 0.44 to 0.85). |
|  | Tanaka et al^116^ | Prospective multicenter study | Investigated the effect of the SGLT2i dapagliflozin on left ventricular (LV) diastolic functional parameters of T2DM patients with stable HF | Patients with T2DM and stable HF | 53 | Dapagliflozin 5 mg | - Administration of dapagliflozin (baseline vs 6 months)   - decreased ratio of mitral inflow (E) to mitral (e′) annular velocities (E/e′) (9.3 vs 8.5 cm/s; p = 0.020),   - improved global longitudinal strain (GLS; 15.5 ± 3.5% vs 16.9 ± 4.1%; p < 0.01), - Improvement of GLS in HF with preserved ejection fraction patients was more significant (from 17.0 ± 1.9% to 18.7 ± 2.0%; p < 0.001) compared to HF with mid-range ejection fraction (from 14.4 ± 2.4% to 15.5 ± 1.8%; p = 0.06) and HF with reduced ejection fraction (from 8.1 ± 1.5% to 7.8 ± 2.1%; p = 0.44) patients. |
| **GLP-1 RA** | Baviera et al^117^ | Real world PS matched study | Efficacy and safety of GLP-1RAs and SGLT2i compared with other AHAs in large and unselected populations of the Lombardy and Apulia regions in Italy. | Patients with T2DM | Lombardy cohort: GLP-1RA, 18 716; SGLT2i, 11 683; Apulia cohort: GLP-1RA, 9772; SGLT2i, 6046 | NA | - Use of GLP-1RAs (vs other AHAs) was associated with lower rates of   - death (HR, 0.61; 95% CI, 0.56 to 0.65, Lombardy; HR, 0.63; 95% CI, 0.55 to 0.71, Apulia), - GLP-1RAs group (vs other AHAs) had reduced occurrence of HF (HR, 0.89, 95% CI, 0.82 to 0.97) in the pooled cohorts. - SGLT2i use (vs other AHAs) decreased risk of   - HF (HR, 0.56, 95% CI, 0.46 to 0.70, Lombardy; HR, 0.57, 95% CI, 0.42 to 0.77, Apulia),   - death (HR, 0.47; 95% CI, 0.40 to 0.54, Lombardy; HR, 0.43, 95% CI, 0.32 to 0.57, Apulia). |
|  | Tonneijck et al^118^ | Post-hoc analyses of four phase-4 clinical intervention trials | Determine effects of GLP-1RA on UA-levels and kidney UA-clearance. | Patients with T2DM | Study A, 9; Study B, 52; Study C, 36; Study D, 35 | NA | - Exenatide-infusion (vs placebo)   - slightly increased plasma-UA (+0.07±0.02mg/dL; P=0.04) and raised absolute-UEUA (+1.58±0.65mg/min/1.73m2; P=0.02) in study A.   - no effect on plasma-UA, but increased fractional-UEUA (+0.76±0.38%, P=0.049) and absolute-UEUA (+0.75±0.27mg/min/1.73m2, P=0.007) in study B.   - no effect on plasma-UA (P=0.8), UEUA (-1.55±0.88%, P=0.09) UENa, or urine-pH (5.52 ± 0.14 vs 5.61 ± 0.15; p = 0.341) in study C.   - increased UENa (0.18 ± 0.05 vs -0.11 ± 0.08; p = 0.003) and urine-pH (5.48 ± 0.18 vs 5.12 ± 0.09; p = 0.013) from baseline, but did not affect plasma-UA or UEUA (p >0.05) in study D. |
|  | Longato et al^119^ | 1:1 PS matched study | Compare CV outcome and AEs of patients with newly added SGLT2i and GLP-1 RA treatment using real-world data | Patients with T2DM | SGLT2i, 4298; GLP-1 RA 4298 | NA | - SGLT2i treatment (vs GLP-1 RA) group experienced lower rate of   - 3P-MACE (HR, 0.68; 95% CI, 0.61 to 0.99; p = 0.043),   - MI (HR, 0.72; 95% CI, 0.53 to 0.98; p = 0.035),   - HHF (HR, 0.59; 95% CI, 0.35 to 0.99; p=0.048),   - hospitalization for CV causes (HR, 0.82; 95% CI, 0.69 to 0.99; p = 0.037). |
|  | Dave et al^120^ | 1:1 PS matched study | Pharmacodynamic effects on CV risk of adding SGLT2i to existing GLP-1 RA therapy | Patients with T2DM | SGLT2i, 12584; sulfonylureas, 12584 | NA | - SGLT2i (vs sulfonylureas) group had lower   - Composite CV end point events (incidence rate per 1000 person; 9.9, 95% CI, 8.1 to 11.9 vs 13.0, 95% CI, 10.9 to 15.3; adjusted pool HR, 0.76; 95% CI, 0.59–0.98),   - event of HHF (incidence rate per 1000 person; 13.0, 95% CI, 11.0 to 15.2 vs 20.8, 95% CI, 18.1 to 23.8; adjusted pool HR, 0.65, 95% CI, 0.50–0.82). |
| **Thiazolidinedione** | Chang et al^121^ | PS matched study | Metabolic and renal function changes with add-on pioglitazone treatment versus basal insulin in patients with T2DM in whom sulfonylurea and metformin regimens failed | Patients with T2DM | Pioglitazone, 559; detemir, 264; glargine, 179 | NA | - Detemir group (vs pioglitazone) had a higher probability of CKD progression (HR, 2.63; 95% CI 1.79 to 3.88). - Glargine treatment (vs pioglitazone) had a higher probability of CKD progression (HR, 3.13; 95% CI 2.01 to 4.87). |
|  | Strongman et al^122^ | PS matched study | Compare the risk of CV and non-CV mortality in patients whose antidiabetic therapy is modified to include pioglitazone compared with an alternative antidiabetic medication at the same stage of disease progression | Patients with T2DM | Pioglitazone exposed 31133; Pioglitazone non-exposed 31133 | NA | - Pioglitazone exposed group (vs pioglitazone non-exposed) had lower crude mortality rate for   - cardiovascular mortality (adjusted HRs, 0.58; 95% CI 0.52 to 0.63; p = 0.012),   - non-cardiovascular mortality (adjusted HRs, 0.63, 95% CI 0.58 to 0.68; p = 0.002). |
|  | Miao et al^123^ |  | Protective effect of pioglitazone against CVD risk among T2DM patients | Patients with T2DM | non‐pioglitazone users, 63 557; pioglitazone users, 8226 | NA | - Pioglitazone (vs non‐pioglitazone) use associated with   - 39% decreased risk of MI (RR, 0.61; 95% CI, 0.42 to 0.90)   - 30% decrease in risk of composite outcome (MI, HF, and stroke) (RR, 0.70; 95% CI,  0.56 to 0.88) |

AHAs = antihyperglycemic agents; CV = cardiovascular; CVD = cardiovascular disease, CI = confidence interval; ; CKD = chronic kidney disease; CVD = cardiovascular disease; DPP-4i = dipeptidyl peptidase-4 inhibitor; GLD = glucose lowering drug; GLP-1 RA = glucagon-like peptide-1 receptor agonists; HF = heart failure; HHF = hospitalization for heart failure; HKD: hospitalization for kidney disease; HR = hazard ratio; KCCQ-TSS = Kansas City Cardiomyopathy Questionnaire total symptom score; LV = left ventricular; MACE = major adverse cardiovascular events; MI = myocardial infraction; NA = not available; PS = propensity-score; RR = relative risk; SGLT-2i = sodium-glucose co-transporter 2 inhibitor; T2DM = type 2 diabetes mellitus; UA = uric acid; UEUA = urinary-excretion of uric acid

**Supplementary Table 4. Meta-analyses describing cardioprotective and nephroprotective properties of major antidiabetic therapies**

| **Drug class** | **Name of Author** | **Objective** | **Population Characteristics** | **Number of Patients** | **Outcome** | |
| --- | --- | --- | --- | --- | --- | --- |
| **SGLT2i** | Zelnikar et al^79^ | Assess reliable estimate of efficacy and safety of SGLT2i in terms of specific outcomes overall and in relevant subgroups | Patients with   - T2DM - established ASCVD‑99% - established ASCVD‑65% - established ASCVD‑40% | 34322 | - SGLT2i treatment reduced   - MACE by 11% (HR, 0.89; 95% CI, 0.83 to 0.96; p = 0.0014), but benefit only seen in patients with atherosclerotic CVD compared to those without (HR, 0.86; 95% CI, 0.80 to 0.93 vs HR, 1.00; 95% CI, 0.87 to 1.16; p for interaction = 0.0501),   - risk of CV death or HHF by 23% (HR, 0.77; 95% CI, 0.71 to 0.84], p<0.0001) in patients with and without atherosclerotic CVD,   - risk of progression of renal disease by 45% (HR, 0.55, 95% CI, 0.48-0.64; p<0.0001) in patients with and without atherosclerotic CVD,   - magnitude of benefit of SGLT2i varied with higher reduction in HHF (p for interaction = 0.0073) and lower in progression of renal disease (p for interaction =- 0.0258). | |
|  | Chewcharat et al^124^ | Assesses efficacy and safety profiles of SGLT-2i for treatment of DM among kidney transplant patients. | Kidney transplant patients with T2DM | 132 | - Compared to baseline, empagliflozin group had reduced   - BMI (WMD = −0.82 kg/m2; 95% CI: −1.41 to −0.24; p = 0.006),   - body weight (WMD = −2.17 kg; 95% CI: −3.20 to −1.15; p<0.001). - Canagliflozin treatment results in decreased   - HbA1C (WMD = −0.87% [95% CI: −1.46 to −0.27; p = 0.004).   - SBP (WMD = −7.15 mmHg 95% CI: −14.27 to −0.03; p = 0.04). - No serious AEs reported, including euglycemic ketoacidosis and acute rejection. | |
|  | Gilbert et al^125^ | Compares the frequency of AKI AEs reports in placebo vs. SGLT2i groups | Patients with T2DM | NA | - Reduction in the likelihood of AKI in SGLT2i vs placebo group (HR, 0.66; 95% CI, 0.54-0.80). | |
|  | Chambergo-Michilot et al^126^ | Assesses effects and safety of SGLT2i in HF patients. | Patients with HF | NA | - SGLT2i treatment reduced   - all-cause mortality (RR, 0.88; 95% CI, 0.79 to 0.98),   - CV mortality (RR, 0.87; 95% CI, 0.77 to 0.99),   - HHF (RR, 0.73; 95% CI, 0.66 to 0.81),   - emergency room visits due to HF (RR, 0.40; 95% CI, 0.21 to 0.76),   - any serious AEs (RR, 0.89; 95% CI, 0.84 to 0.94),   - SBP (MD, −0.70; 95% CI −0.73 to −0.68)   - weight (MD, −0.81; 95% CI, −0.82 to −0.80) - SGLT2i treatment increased   - hematocrit (MD, 1.38; 95% CI, 0.02 to 2.74)   - creatinine (MD, 2.65; 95% CI, 2.63 to 2.67). | |
|  | Salsali et al^127^ | Assesses the effect of empagliflozin (10 and 25 mg daily once) on CV risk in patients with T2DM. | Patients with T2DM | Empagliflozin: 7457;  Placebo: 3835 | - Empagliflozin (vs placebo) reduced the risk of   - 4‐point MACE (HR, 0.86; 95% CI, 0.76 to 0.98),   - 3-point MACE (HR, 0.84; 95% CI, 0.73 to 0.96). | |
|  | Tang et al^128^ | Compares CV safety and mortality risk associated with the use of SGLT2i | Patients with T2DM | 29859 | - Empagliflozin (vs placebo) was associated with lower risk of   - all-cause mortality (OR, 0.67; 95% CI, 0.56 to 0.81),   - major adverse CV events (OR, 0.81; 95% CI 0.70 to 0.93). | |
|  | Malik et al^129^ | Evaluates SGLT2i CV effects in patients with T2DM and CKD stage 3 or higher. | Patients with   - T2DM - CKD | 6527 | - SGLT2i lowered the risk of   - hospitalization due to exacerbation of HF (HR, 0.61; 95% CI, 0.47 to 0.77; p<0.01),   - MI (HR, 0.78; 95% CI, 0.62 to 0.97; p = 0.03),   - stroke (HR, 0.83; 95% CI, 0.62 to 1.11; p = 0.21),   - composite outcome of MACE (HR, 0.80; 95% CI, 0.69 to 0.92; p<0.01). - Canagliflozin reduced incidence of MI, stroke, and risk of hospitalization due to exacerbation of HF. | |
|  | Usman et al^130^ | Assess the CV effects of SGLT2i. |  | 34987 | - SGLT2i treatment reduced   - all-cause mortality (OR, 0.79; 95% CI, 0.70 to 0.89; p< 0.001),   - major adverse cardiac events (OR, 0.8; 95% CI 0.76 to -0.92; p< 0.001),   - non-fatal MI (OR, 0.85; 95% CI 0.73 to 0.98; p = 0.03),   - HF/HHF (OR, 0.67; 95% CI, 0.59 to 0.76; p< 0.001). | |
|  | Zannad et al^131^ | Estimate the effect of SGLT-2 inhibition on fatal and non-fatal HF events and renal outcomes in all randomly assigned patients with HFrEF. | Patients with   - HFrEF - With or without DM | 8474 | - Estimated treatment effect resulted in   - 13% reduction in all-cause death (pooled HR, 0·87; 95% CI, 0·77 to 0·98; p = 0·018),   - 14% reduction in cardiovascular death (pooled HR, 0·86, 95% CI, 0·76 to 0·98; p = 0·027). - SGLT2i users had   - reduced relative combined risk of CV death or first HHF (26%; pooled HR, 0·74; 95% CI, 0·68 to 0·82; p<0·0001).   - reduced risk of the composite renal endpoint (pooled HR, 0·62; 95% CI, 0·43 to 0·90; p = 0.013)   - decrease in the composite of recurrent HHF or CV death (25%; pooled HR, 0·75; 0·68 to 0·84; p<0·0001). | |
|  | Ryan et al^132^ | Examines the comparative effectiveness within the SGLT2i class and non‐SGLT2i antihyperglycemic agents | Patients with T2DM | NA | - Canagliflozin group (vs non‐SGLT2i) had reduced risk of HHF (HR, 0.39; 95% CI, 0.26 to 0.60). | |
|  | McGuire et al^80^ | CV and kidney outcome of all 4 available SGLT2i in patients with T2DM | Patients with   - T2DM - Atherosclerotic CV disease | 46969 | - Overall use of SGLT2i associated with reduced risk of   - MACE (HR, 0.90; 95% CI, 0.85 to 0.95; p = 0.27),   - HHF/CV death (HR, 0.78; 95% CI, 0.73 to 0.84; p= 0.09),   - kidney outcomes (HR, 0.62; 95% CI, 0.56 to 0.70; p = 0.09) | |
|  | Arnott et al^133^ | CV benefits and the effects on key safety outcomes of SGLT2i | Patients with T2DM | 38723 | - SGLT2i- (vs placebo) was associated with an overall proportional reduction in   - MACE (12%, HR, 0.88; 95% CI, 0.82 to 0.94; p<0.001),   - CV death (17%; HR, 0.83; 95% CI, 0.75 to 0.92; p<0.001),   - MI (HR, 0.88; 95% CI, 0.80 to 0.97; p = 0.01),   - HHF (HR, 0.68; 95% CI, 0.60 to 0.76; p<0.001),   - death from any cause (HR, 0.85; 95% CI, 0.79 to 0.92; p<0.001). | |
|  | Giugliano et al^134^ | Determine extent of MACE prevention with benefits increasing in more sever kidney disease | Patients with T2DM | 38724 | - SGLT2i- (vs placebo) group had   - reduced risk of MACE by 12% (HR, 0.88; 95% CI 0.82 to 0.94; p < 0.001), - greater proportional risk reduction for MACE in patients with reduced kidney function than patients with preserved kidney function (HR, 0.77; 95% CI 0.65 to 0.90 vs HR, 0.91; 95% CI 0.85 to 0.99; p for heterogeneity between subgroups= 0.053) | |
| **GLP-1 RA** | Alfayez et al^135^ | Assess GLP-1RAs and DPP-4i CV safety in patients with T2DM | Patients with T2DM | NA | - GLP-1RAs (vs placebo) associated with reduction in the odds of   - major adverse CV events (13%, OR, 0.87; 95% CI, 0.79 to 0.96; p = 0.04)   - CV death o (12%; OR, 0.88; 95% CI, 0.8 to 0.97; p = 0.008),   - death from any cause (11%; OR, 0.89; 95% CI, 0.82 to 0.96; p = 0.002)   - stroke (13 %; OR, 0.87; 95% CI, 0.77 to 0.98; p = 0.02). | |
|  | Wu et al^136^ | Comparison of CV safety of incretin-based therapies in patients with T2DM | Patients with T2DM | 180,000 | - GLP-1RA group had reduction in the risk of CV events compared to placebo (OR 0.89, 95%CI: 0.80-0.99) and sulfonylurea (OR 0.76, 95%CI: 0.59 to 0.99) groups. | |
|  | Decembrini et al^137^ | Assesses the effects of GLP-1RA on nephropathy | Patients with T2DM | GLP1-RA: 11,399; comparator: 10,114 | - GLP1-RA reduced the incidence of nephropathy compared to comparators (MH-OR, 0.74; 95% CI, 0.60 to 0.92; p = 0.005). | |
|  | Mannucci et al^77^ | Assesses effects of GLP-1RAs on MACE | Patients with T2DM | GLP-1RA: 2937;  Control: 3309 | - GLP-1RA treatment associated with a reduction in   - MACE (MH-OR, 0.87; 95% CI 0.81 to 0.93),   - cardiovascular mortality (MH-OR, 0.88; 95% CI, 0.80 to 0.96),   - all-cause mortality reduced (MH-OR, 0.90; 95% CI, 0.82 to 0.98),   - myocardial infarction (MH-OR, 0.91; 95% CI 0.84 to 0.98)   - stroke (MH-OR, 0.86; 95% CI, 0.77 to 0.97). | |
|  | Giugliano et al ^138^ | Examines the overall effect of GLP-1RAs on cardiorenal efficacy | Patients with T2DM | 56 004 | - GLP-1RA reduced   - MACE by 13% (HR, 0.87; 95% CI, 0.80 to 0.96; p = 0.011) in patients with and without CVD.   - risks of HHF (HR, 0.91, 95% CI, 0.86 to 0.97)   - all-cause mortality (HR, 0.89, 95% CI, 0.79 to 0.99).   - risk of cardiovascular death by 12%, non-fatal stroke by 16%, HHF by 9%, all-cause mortality by 11% and broad composite kidney outcome by 17%. | |
|  | Yamada et al^139^ | Benefits of SGLT-2 inhibitors and GLP-1 RAs in CKD patients | Patients with T2DM | 32949 | - SGLT2i group (vs placebo) had reduced risk of   - 3- point MACE events (RR, 0.85; 95% CI, 0.75 to 0.96),   - Renal events (RR, 0.68; 95% CI, 0.59 to 0.78). - SGLT2i treatment (vs GLP-1RA) was associated with decreased risk of renal events (RR, 0.79; 95% CI, 0.63 to 0.99). - GLP-1 analogues (vs placebo) lowered MACE-3 events (RR, 0.81; 95% CI, 0.69 to 0.95). | |
|  | McKee et al^140^ | Assist in the prescribing decision regarding severity of illness and risk for adverse events | Patients with T2DM | GLP-1 RA, 51762; SGLT2i, 33457 | - SGLT2i (vs control) group showed   - improvement in MACE (RR, 0.87; 95% CI, 0.82 to 0.93; p < 0.001),   - improvement in HHR (RR, 0.68; 95% CI, 0.61 to 0.76; p < 0.001),   - reduction in renal outcome (RR, 0.67; 95% CI, 0.57 to 0.79; p < 0.001), - GLP-1 RA (vs control) group showed   - improvement in MACE (RR, 0.91; 95% CI, 0.87 to 0.96; p < 0.001),   - reduction in renal outcome (RR, 0.83; 95% CI, 0.75 to 0.91; p ≤ 0.001). | |
|  | Sattar et al^83^ | Evidence on the cardiovascular benefits and risks of GLP-1 RAs from outcome trials in patients with T2DM | Patients with T2DM | 60080 | - Overall, GLP-1 RA reduced   - MACE by 14% (HR, 0.86; 95% CI, 0.80 to 0.93; p<0.0001), with no significant heterogeneity across GLP-1 RA structural homology or eight other examined subgroups (all p interaction ≥0.14),   - all-cause mortality by 12% (HR, 0.88; 95% CI, 0.82 to 0.94; p=0.0001),   - hospital admission for HF by 11% (HR, 0.89; 95% CI 0.82 to 0.98; p=0.013),   - composite kidney outcome by 21% (HR, 0.79, 95% CI, 0.73 to 0.87; p<0.0001), - There was no increase in risk of severe hypoglycaemia, retinopathy, or pancreatic adverse effects of GLP-1 RA. - In sensitivity analyses (excluding ELIXA study), all benefits marginally increased, including the outcome of worsening of kidney function, based on eGFR change (HR, 0.82; 95% CI 0.69 to 0.98; p=0·030). | |
| **Thiazolidinedione** | Zhou et al^141^ | Evaluates the effect of pioglitazone on the primary and secondary prevention of CVDs and renal AEs in patients with or at high risk of T2DM | Patients   - with T2DM - at higher risk of T2DM | 19645 | - Pioglitazone treatment   - reduced risk of MACE (RR, 0.8; 95% CI, 0.7 to 0.9), nonfatal MI (RR, 0.8 95% CI, 0.6 to 1.0) and nonfatal stroke (RR, 0.8; 95% CI, 0.7 to 0.9) in patients with a history of established CVDs   - increased the risk of HHF (RR, 1.3; 95% CI, 1.1 to 1.6).   - reduced albuminuria by 18.5% (WMD = 18.5%; 95% CI, 21.1 to 16.0) in patients with different renal function categories. | |
|  | Liao et al^142^ | Evaluates the effect of pioglitazone in people with IR, pre-diabetes and T2DM | Patients with   - IR - pre-diabetes - T2DM | 12026 | - Pioglitazone use associated with   - lower risk of MACE in patients with pre-diabetes or IR (RR, 0.77; 95% CI 0.64 to 0.93), and diabetes (RR 0.83, 95% CI 0.72 to 0.97).   - higher risk of HR (RR, 1.32; 95% CI, 1.14 to 1.54), edema (RR, 1.63; CI 1.52 to 1.75) and weight gain (RR, 1.60; CI 1.50 to 1.72). |  |
|  | de Jong et al^143^ | Assesses the effects of pioglitazone treatment on the secondary prevention of CVD | Patients with   - CVD - with or without T2DM | 10252 | - Pioglitazone reduced recurrent MACE (RR, 0.74; 95% 0.60 to 0.92), MI (RR, 0.77; 95% CI 0.64 to 0.93), or stroke (RR, 0.81; 95% CI 0.68 to 0.96). - pioglitazone use associated with an increased risk of HF (RR, 1.33; 95% CI, 1.14 to 1.54). | |
|  | Lee et al^144^ | Evaluates the effect of pioglitazone therapy in reducing the risk of recurrent stroke in stroke patients | Stroke patients with   - T2DM, - Prediabetes - IR | 4980 | - Pioglitazone use was associated with lower risk of   - recurrent stroke (HR, 0.68; 95% CI, 0.50 to 0.92; p = 0.01),   - future major vascular events (HR, 0.75; 95% CI, 0.64 to 0.87; p = 0.0001) | |
|  | Sinha and Ghosal^145^ | Address the various points of debate regarding pioglitazone use | Patients with T2DM | Pioglitazone: 5452; Controls 5525 | - Pioglitazone users (vs active control/placebo) had reduced odds of   - MACE (14%; MH-OR, 0.86; 95% CI 0.75 to 0.98),   - stroke (23%; MH-OR, 0.77; 95% CI 0.60 to 0.99). - Pioglitazone treated group had increased odds of   - HF (MH-OR, 1.47; 95% CI 1.26 to 1.71)   - HHF (MH-OR, 1.48; 95% CI 1.21 to 1.81). | |

AE = adverse event; AKI = acute kidney injury; BMI = body mass index; CI = confidence interval; CKD = chronic kidney disease; CV = cardiovascular; CVD = cardiovascular disease; DM = diabetes mellitus; DPP-4i = dipeptidyl peptidase-4 inhibitor; eGFR = estimated glomerular filtration rate; GLP-1 RA = glucagon-like peptide-1 receptor agonists; HbA_1C_ = Glycated hemoglobin; HF = heart failure; HHF = hospitalization for heart failure; HR = hazard ratio; IR = insulin resistance; MACE = major adverse cardiovascular events; MI = myocardial infraction; MD = mean differences; MH-OR = Mantel–Haenszel odds ratio; OR = odds ratio; NA = not available; RR = risk ratio; SGLT-2i = sodium-glucose co-transporter 2 inhibitor; SBP = systolic blood pressure; T2DM = type 2 diabetes mellitus; WMD = weighted mean difference
